# Vision-Related Patient-Reported Outcomes in Randomized Controlled Trials of Age-Related Macular Degeneration: A Systematic Review

**DOI:** 10.1016/j.xops.2026.101308

**Published:** 2026-06-30

**Authors:** Nicolas S. Bodmer, Melisa Guezelguen, Leyla Huber, Jeremy Howell, Livia Faes, Lucas M. Bachmann, Dun Jack Fu, Kimberly Spooner, Sobha Sivaprasad, Martin K. Schmid

**Affiliations:** 1 Institute of Epidemiology, Biostatistics and Prevention, University of Zurich, Zurich, Switzerland; 2 Institute of Health Policy, Management and Evaluation (IHPME), University of Toronto, Toronto, Canada; 3 Medical Faculty, University of Zurich, Zurich, Switzerland; 4 Medical Faculty, University of Basel, Basel, Switzerland; 5 Department of Ophthalmology, Cantonal Hospital of Lucerne, Lucerne, Switzerland; 6 Department of Quantitative Biomedicine, University of Zurich, Zurich, Switzerland; 7 Research Department, Medignition Inc, Zurich, Switzerland; 8 Ophthalmology Department, Cantonal Hospital Winterthur, Winterthur, Switzerland; 9 NHS Foundation Trust, Moorfields Eye Hospital, London, United Kingdom

**Keywords:** Age-related macular degeneration, Patient-reported outcome measures, Systematic review.

## Abstract

**Topic:**

This systematic review examined how vision-related patient-reported outcome measures (PROMs) were selected, operationalized, and reported at baseline in randomized controlled trials (RCTs) enrolling patients across the spectrum of age-related macular degeneration (AMD).

**Clinical Relevance:**

Age-related macular degeneration is a major cause of visual disability, and visual acuity alone does not fully capture its effects on everyday functioning and quality of life. Inconsistent selection and incomplete reporting of PROMs may limit interpretation, comparison across trials, and evidence synthesis.

**Methods:**

A systematic search of Medical Literature Analysis and Retrieval System Online, Embase, Web of Science Core Collection, and Scopus was conducted from database inception to September 27, 2023, supplemented by a targeted update for relevant 2024 and 2025 publications. Eligible studies were RCTs enrolling ≥20 participants with AMD and reporting both baseline visual acuity and baseline vision-related PROM data. Data on PROM instrument selection, scoring, baseline distributions, laterality context, and subgroup reporting were extracted and synthesized descriptively.

**Results:**

Thirty-eight RCTs met the inclusion criteria, spanning early through late-stage AMD. Across included trials, 11 distinct PROM instruments were reported. The National Eye Institute Visual Function Questionnaire family predominated (78.9%), with the National Eye Institute Visual Function Questionnaire‑25 being the most commonly used version (n = 17). Baseline patient-reported functional status varied by disease stage: early and intermediate AMD cohorts reported higher scores (75.3–89.1), whereas trials enrolling neovascular AMD patients showed lower and more heterogeneous scores (59.1–77.9). Subscale and domain reporting was inconsistent, with many trials presenting only composite scores or a limited subset of subscales. Baseline PROM results were not reported by subgroup, preventing comparisons across patient populations. Reporting of visual function was incomplete with only 13% of studies reporting both better-seeing and worse-seeing eyes.

**Conclusion:**

The interpretive value of PROMs in AMD trials is constrained by inconsistent baseline reporting and limited reporting of both-eye and binocular acuity needed to contextualize patient-level function. Enhanced consistency in stage-appropriate PROM selection and routine reporting of baseline distributions are essential to support patient-centered evaluation and evidence synthesis in AMD research.

**Financial Disclosure(s):**

Proprietary or commercial disclosure may be found in the Footnotes and Disclosures at the end of this article.

Age-related macular degeneration (AMD) is a leading cause of irreversible vision loss worldwide, with an estimated 8.06 million individuals affected by AMD-related visual impairment in 2021, a figure projected to rise to 21.34 million by 2050.^1^ However, this epidemiologic burden is incompletely captured by high-contrast visual acuity, the dominant endpoint in most clinical trials and regulatory pathways. Patients with early or intermediate AMD may experience functionally meaningful deficits in contrast sensitivity, low-luminance vision, and dark adaptation despite relatively preserved best-corrected visual acuity (BCVA).^2^, ^3^, ^4^ In late AMD, macular exudation, fibrosis, and atrophy can additionally produce scotomas and metamorphopsia, and patients with central retinal damage may adopt eccentric preferred retinal loci with variably stable fixation, further limiting everyday activities and reducing vision-related quality of life.^5^^,^^6^ The multidimensional impact of AMD on reading, mobility, independence, and psychosocial well-being reveals a persistent gap between what is typically quantified in clinical testing and what patients experience in daily life.^5^

Consequently, patient-reported outcome measures (PROMs) have gained prominence as complementary endpoints, providing standardized assessments of patient-perceived symptoms, functional limitations, and well-being, and are increasingly expected in comprehensive evaluations of therapeutic interventions.^7^ This expectation reflects regulatory emphasis on patient-centered evidence and value-based health care initiatives that promote standardized outcome sets incorporating PROMs.^8^, ^9^, ^10^ In ophthalmology, and specifically in AMD, the International Consortium for Health Outcomes Measurement macular degeneration standard set explicitly includes PROMs as part of a minimum core data set, positioning vision-related quality of life measurement as central to benchmarking and value-based assessment.^11^ In AMD research, the National Eye Institute Visual Function Questionnaire (NEI VFQ) family is the most frequently used PROM for patient-reported vision-related functioning.^12^ In pivotal neovascular age-related macular degeneration (nAMD) trials, NEI VFQ-25 scores improved with antiangiogenic treatment across multiple subscales and the composite score, supporting responsiveness of vision-specific PROMs to treatment effects.^13^

Despite rising expectations for patient-centered measurement, PROM implementation and reporting in AMD trials may not fully align with contemporary methodological standards. A 2015 systematic review found that PROMs were included in <15% of AMD randomized controlled trials (RCTs), with the NEI VFQ-25 predominating (>60% of PROMs-inclusive studies).^14^ Since then, the therapeutic landscape and methodological standards for trial outcome reporting have evolved substantially. It remains unclear whether contemporary AMD RCTs report PROMs with sufficient consistency and transparency to support cross-study comparability, valid interpretations, or whether the evidence base supports synthesis of patient-reported functioning across interventions, populations, and disease stages.

We therefore conducted a systematic review of AMD RCTs incorporating vision-related PROMs to characterize the instruments used, their operationalization, and the completeness of baseline PROM reporting. We specifically assessed whether baseline PROM results were reported with sufficient context to support interpretation across AMD stage, treated-eye and fellow-eye status, better-seeing eye (BSE) and worse-seeing eye (WSE) status, binocular visual function, geography, and ethnicity. This mapping identifies reporting gaps that limit the interpretability of PROMs in AMD trials and informs priorities for more consistent patient-centered outcome assessment.

## Methods

This systematic review was conducted and reported in accordance with the Preferred Reporting Items for Systematic Reviews and Meta-Analyses 2020 statement and prospectively registered on the International Prospective Register of Systematic Reviews (CRD42023435112).^15^ A separate review protocol was not prepared.

### Search Strategy

We conducted a comprehensive literature search across Medical Literature Analysis and Retrieval System Online, Embase, Web of Science Core Collection, and Scopus from database inception through September 27, 2023. To capture recently published evidence, we additionally performed a targeted supplementary search for relevant 2024 and 2025 publications using the same eligibility criteria. This update included targeted searches of bibliographic databases, trial registries, regulatory documents, reference lists of included studies, and secondary publications from known AMD trial programs. Candidate records identified through this process were screened against the same inclusion and exclusion criteria as records identified in the original database search. The full electronic search strategies for all databases, including search terms, filters, and limits, are provided in Supplementary Data 1 (Tables S1 and S2, available at www.ophthalmologyscience.org).

### Selection of Studies

We included peer-reviewed publications of RCTs reporting original patient data from ≥20 randomized participants with AMD and using a vision-related PROM. The threshold of 20 randomized participants was prespecified as a pragmatic minimum to exclude very small feasibility or pilot reports with unstable baseline PROM distributions while retaining smaller low-vision, rehabilitation, and nonpharmacological AMD trials that could contribute relevant information on PROM use and reporting. This threshold was not intended as a formal statistical power criterion. Eligible publications were required to report extractable baseline visual acuity and baseline vision-related PROM data for the AMD population or AMD subgroup. We required baseline PROM data because the objective of this review was to characterize patient-reported functional status at trial entry and to assess whether baseline reporting was sufficiently complete to contextualize subsequent PROM change. Follow-up values or change scores alone were not considered adequate because they cannot reliably reconstruct baseline patient-reported function. The original International Prospective Register of Systematic Reviews registration intended to include both randomized and observational studies. During review conduct, we restricted eligibility to RCTs because observational studies of PROM use in AMD were inconsistently indexed and could not be retrieved exhaustively with sufficient reproducibility. This amendment was made to preserve a transparent and reproducible evidence base. No restrictions were applied regarding language or study quality (detailed eligibility criteria: Supplementary Data 2, available at www.ophthalmologyscience.org).

After deduplication, references were imported into Covidence and independently screened at title, abstract, and full-text stages by trained reviewers (N.S.B., M.G., L.H., J.H., L.F., D.J.F., K.S.), with disagreements resolved by consensus.^16^

### Data Extraction

Reviewers independently extracted data in duplicate using a predesigned, pilot-tested form. Discrepancies were resolved by discussion. Extracted variables included study characteristics (author, year, country), selection criteria, participant demographics, baseline BCVA (BSE, WSE, study and fellow eye), PROM instrument details (name, description, operationalization, baseline results), and subgroup analyses by geography and ethnicity.

### Assessment of Methodological Quality

We assessed the integrity of baseline PROM data using selected domains of the Cochrane Risk of Bias 2 tool adapted for baseline, preintervention data (Supplementary Data 3, available at www.ophthalmologyscience.org).^17^ Because this review focused on cross-sectional baseline characteristics rather than treatment effects, we restricted the assessment to 3 domains relevant to the integrity of baseline data: (1) randomization process (sequence generation and allocation concealment), (2) completeness of baseline outcome data, and (3) outcome measurement. In this context, “high risk” in the randomization domain indicates potential selection bias that may compromise the representativeness of the baseline cohort. “High risk” in the missing data domain indicates that baseline PROM values were unavailable for a substantial proportion of randomized participants, which may bias the reported functional status if missingness is related to disease severity or patient characteristics. Two independent reviewers (N.S.B., L.F.) applied the tool to all included studies, with disagreements resolved by consensus.

### Data Analysis and Synthesis

Means and standard deviations were extracted for continuous variables. When studies reported subgroup data only, pooled study-level means and standard deviations were calculated using standard sample size–weighted formulas. Nonparametric data (medians, interquartile ranges) were retained in their original format and reported as such.

Patient-reported outcome measure usage frequencies and operationalization (instrument version, scoring, and reporting approach) were compared across studies. Subgroup results stratified by geography and ethnicity were summarized descriptively where data were available. Between-study variation in baseline PROM scores was examined descriptively to explore differences in self-reported visual function by trial population and setting. To assess changes in PROM use over time, trials were stratified by publication era (pre-2015 vs. 2015 onward). No sensitivity analyses, formal assessment of reporting bias, or certainty assessment of the body of evidence were performed because no quantitative synthesis was performed.

## Results

### Study Selection

**Figure 1:**
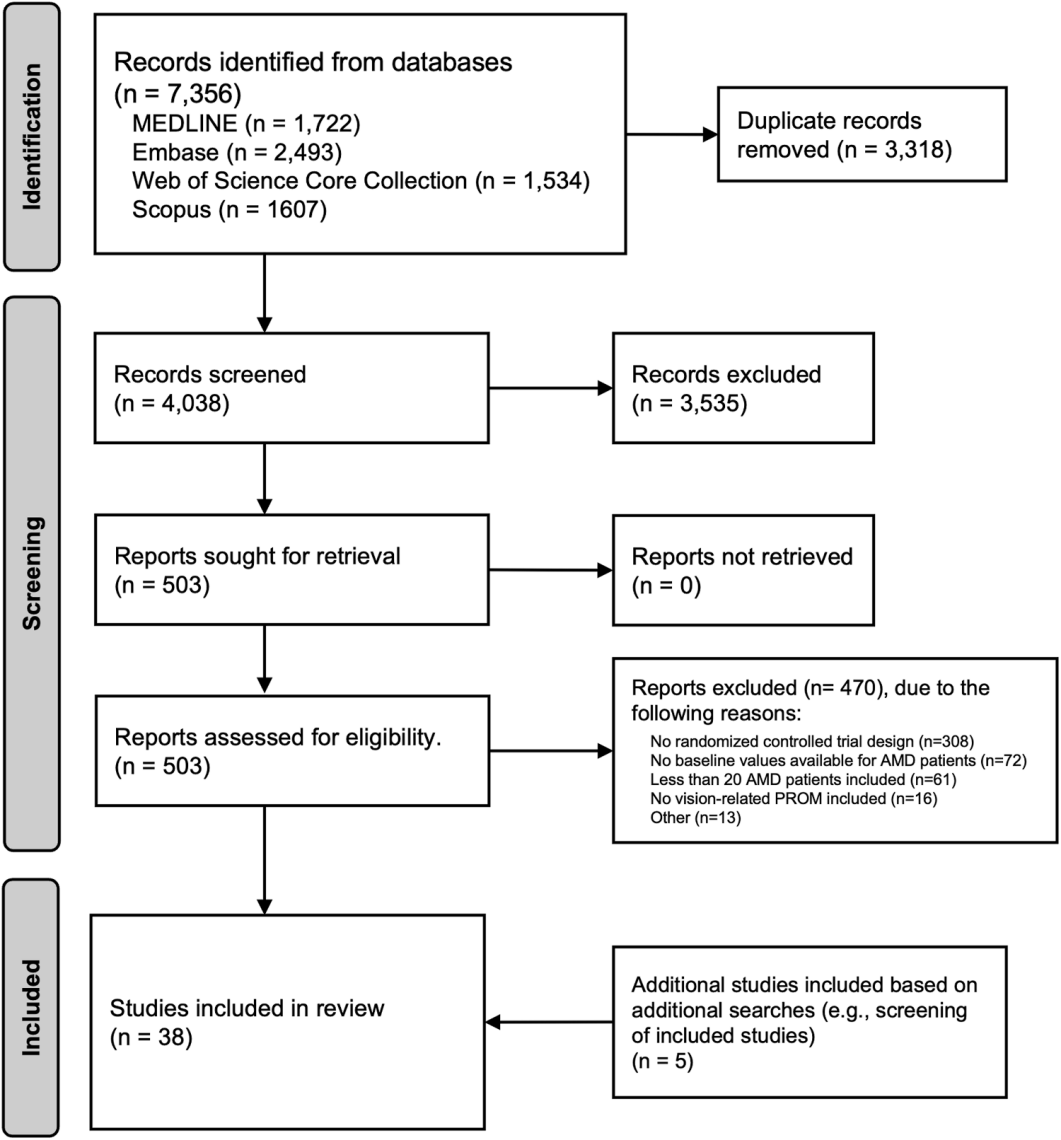
Preferred Reporting Items for Systematic Reviews and Meta-Analyses 2020 flow diagram of study identification, screening, eligibility assessment, and inclusion in the systematic review of vision-related patient-reported outcome measures in randomized controlled trials of AMD. Thirty-eight randomized controlled trials met the inclusion criteria, including 33 identified through database searches and 5 through supplementary searches. AMD = age-related macular degeneration; MEDLINE = Medical Literature Analysis and Retrieval System Online; PROM = patient-reported outcome measure.

Database searches identified 7356 records. After deduplication and title/abstract screening, 503 reports were assessed at the full-text stage, of which 38 RCTs met the inclusion criteria (Fig 1).^18^, ^19^, ^20^, ^21^, ^22^, ^23^, ^24^, ^25^, ^26^, ^27^, ^28^, ^29^, ^30^, ^31^, ^32^, ^33^, ^34^, ^35^, ^36^, ^37^, ^38^, ^39^, ^40^, ^41^, ^42^, ^43^, ^44^, ^45^, ^46^, ^47^, ^48^, ^49^, ^50^, ^51^, ^52^, ^53^, ^54^, ^55^, ^56^ Five of the 38 included studies were identified through supplementary searches, including screening of reference lists of included studies. Among the 503 reports assessed for eligibility, 470 were excluded for the following reasons: no RCT design (n = 308), no extractable baseline values available for AMD patients (n = 72), <20 AMD patients included (n = 61), no vision-related PROM included (n = 16), or other reasons (n = 13). Several large-scale RCTs were excluded because baseline PROM values were not reported and could neither be identified in trial registries or regulatory documentation nor reconstructed from figures, reported change scores, or follow-up data. For example, the Age-Related Eye Disease Study, although a landmark randomized trial in AMD, was not eligible because the NEI VFQ data reported in Age-Related Eye Disease Study Report No. 10 were collected at the 5-year clinic visit rather than at randomized trial baseline.^57^

The included trials spanned publication years 2001 to 2024 and recruited participants across North America, Europe, Asia, and Oceania, with several large multinational phase III programs enrolling across multiple countries, including trials recruiting across 23, 27, and 32 countries, respectively (Table 1). Sample sizes varied widely, from 20 to approximately 2500 participants, ranging from small single-center trials to large multinational phase III therapeutic trials.

**Table 1:** Study Characteristics

Author, yr	Trial Name (Abbreviation)	Country/Region	AMD Classification	Patients Randomized
Brody, 2001	n.r.	United States	AMD, not specified	151
Dong, 2004	Submacular Surgery Trial (SST)	United States	Exudative neovascular AMD	789 (group B + N)
Maguire, 2004 + Ying, 2008	Complications of Age-Related Macular Degeneration Prevention Trial (CAPT)	United States	Intermediate AMD	1052
Reeves, 2004	n.r.	England	AMD, not specified	226
Brody, 2005	n.r.	United States	AMD, not specified	252
Smith, 2005	n.r.	England	AMD, not specified	243
Stevenson, 2005	Subfoveal Radiotherapy Study (SFRADS)	United Kingdom	Exudative neovascular AMD	203
Chang, 2007	Minimally Classic/Occult Trial of the Anti-VEGF Antibody Ranibizumab in the Treatment of Neovascular Age-Related Macular Degeneration (MARINA)	United States	Exudative neovascular AMD	716
Lamoureux, 2007	The Cataract and Age-Related Maculopathy Study	Australia	Early and intermediate AMD	60
Lüke, 2007	Full macular translocation plus photodynamic therapy (FMT-PDT)	Germany	Exudative neovascular AMD	50
Rovner, 2007	Preventing Depression in AMD Trial	United States	Exudative neovascular AMD	206
Leys, 2008	Vascular Endothelial Growth Factor Inhibition Study in Ocular Neovascularization (VISION)	Multinational (United States, Canada, Europe, Israel, Australia, and South America)	Exudative neovascular AMD	578
Bressler, 2009	Anti-VEGF Antibody for the Treatment of Predominantly Classic Choroidal Neovascularization in Age-Related Macular Degeneration (ANCHOR)	Multinational (United States, Australia, Czech Republic, France, Germany, Hungary)	Exudative neovascular AMD	423
Odergren, 2010	n.r.	Sweden	Exudative neovascular AMD	98
Richer, 2011	Zeaxanthin and Visual Function study (ZVF)	United States	Early and intermediate AMD	60
Heier, 2012	VEGF Trap-Eye: Investigation of Efficacy and Safety in Wet AMD 1 and 2 (VIEW 1 + 2)	VIEW 1: United States, Canada VIEW 2: Europe, the Middle East, the Asia-Pacific region, Latin America	Exudative neovascular AMD	2457
Piermarocchi, 2012	Carotenoids in Age-related Maculopathy Italian Study (CARMIS)	Italy	Intermediate AMD	145
Brunner, 2013	n.r.	Austria	Dry AMD (unclear if non-neovascular)	54
Mielke, 2013	n.r.	Germany	AMD, not specified	20
Rovner, 2013	Improving Function in Age-Related Macular Degeneration (IF-AMD)	United States	Exudative neovascular AMD and geographic atrophy (late-stage AMD)	241
Rovner, 2014	VITAL (Low Vision Depression Trial)	United States	Exudative neovascular AMD and geographic atrophy (late-stage AMD)	188
Huang, 2015	n.r.	China	Early and intermediate AMD	112
Markun, 2015	Chronic Care for Wet Age-Related Macular Degeneration (CHARMED)	Switzerland	Exudative neovascular AMD	169
Riazi, 2016	n.r.	Iran	Dry AMD	69
Tao, 2016	n.r.	China	Dry AMD	100
Akuffo, 2017	Central Retinal Enrichment Supplementation Trials: AMD report (CREST AMD)	Ireland	Early and intermediate AMD	121
Lek, 2017	Laser intervention in Early stages of Age-related macular Degeneration (LEAD)	Australia, Northern Ireland	Intermediate AMD	292
Lee, 2018	Intravitreal aflibercept for polypoidal choroidal vasculopathy (PLANET)	Multinational (Australia, Germany, Hong Kong, Hungary, Japan, Singapore, South Korea, Taiwan)	Exudative neovascular AMD	318
Kaltenegger, 2019	n.r.	Germany	AMD, not specified/geographic atrophy	37
Heier, 2020	CHROMA + SPECTRI	Multinational (23 countries)	Geographic atrophy (late-stage AMD)	1881
Kunimoto, 2020	CEDAR + SEQUOIA	Multinational (32 countries)	Exudative neovascular AMD	1888
Visser, 2020_1	n.r.	Netherlands	Exudative neovascular AMD	191
Visser, 2020_2	E-Scoop Trial	Netherlands	AMD, not specified	185
Gabrielle, 2023	Surgery, Tissue Plasminogen Activator, Antiangiogenic Agents, and Age-Related Macular Degeneration Study (STAR∗)	France	Exudative neovascular AMD	90
Tanveer, 2023	n.r.	Pakistan	AMD, not specified	88
Boyer, 2024	Lightsite III	United States	Dry AMD	100
Jackson, 2024	StereoTactic radiotherapy for wet Age-Related macular degeneration (STAR∗)	United Kingdom	Exudative neovascular AMD	411
Lanzetta, 2024	PULSAR	Multinational (27 countries)	Exudative neovascular AMD	1011

AMD = age-related macular degeneration; n.r. = not reported.

∗ Same abbreviations but different trials.

### Characterization of Study Populations

Across the 38 included trials, eligibility criteria spanned the full AMD severity spectrum. Trials enrolling nAMD only were most common (16/38, 42.1%), followed by trials enrolling early and/or intermediate nonexudative AMD (7/38, 18.4%), dry AMD not further specified (4/38, 10.5%), other mixed AMD populations spanning >1 phenotype or stage (3/38, 7.9%), and geographic atrophy only (1/38, 2.6%). In 7/38 trials (18.4%), AMD stage was not specified. Phenotyping specificity improved over time, with trials classified as “AMD, not specified” decreasing from 5/21 (23.8%) before 2015 to 2/17 (11.8%) from 2015 onward.

### Visual Acuity Reporting by Eye and Binocular Outcomes

Across the 38 included studies, BSE BCVA was the most commonly reported metric (11/38, 29%), with 6 studies reporting BSE only and 5 reporting both BSE and WSE acuity. Worse-seeing eye acuity was available in 7/38 studies (18%). Three studies additionally reported fellow-eye BCVA alongside the study-eye outcomes (3/38, 8%). Binocular function outcomes were rarely reported (1/38, 3%).

### Patient-Reported Outcome Measure Landscape

Across the 38 included RCTs, 11 distinct vision-related PROM instruments were reported (Table 2). The NEI VFQ group predominated, appearing in 78.9% (30/38) of studies. Within this group, the NEI VFQ-25 was the most frequent version (17 studies), followed by the NEI VFQ-39 (6 studies) and the NEI VFQ-25 supplemented with 6 additional appendix items (3 studies). Other NEI VFQ variants, including shortened versions (NEI VFQ-17) or those where the specific version or the adaptation was not further specified, were used only sporadically. Among non-NEI VFQ instruments, the Impact of Vision Impairment questionnaire was the most commonly reported (n = 3). Low-luminance or night-vision–focused measures, such as the Night Vision Questionnaire-10, was used in 2 studies, as was the Melbourne Low-Vision Activities of Daily Living Index. All remaining instruments were each reported in a single trial. A temporal shift was observed in instrument selection. Trials published before 2015 used a wider array of vision-specific PROMs, whereas post-2015 trials predominantly used NEI VFQ.

**Table 2:** PROMs Used in Included Studies

PROM Name	Frequency (n Studies)	Studies
National Eye Institute Visual Function Questionnaire (NEI VFQ)		
NEI VFQ-25	17	Maguire (2004) + Ying (2008); Smith (2005); Leys (2008); Odergren (2010); Richer (2011); Piermarocchi (2012); Mielke (2013); Heier (2012); Huang (2015); Markun (2015); Akuffo (2017); Lee (2018); Kunimoto (2020); Gabrielle (2023); Boyer (2024); Jackson (2024); Lanzetta (2024)
NEI VFQ-39	6	Brody (2001); Dong (2004); Lüke (2007); Rovner (2013); Visser (2020_1); Visser (2020_2)
NEI VFQ-25 + 6 appendix items	3	Chang (2007); Bressler (2009); Heier (2020)
NEI VFQ-25‑specific subscales only	1	Rovner (2014)
NEI VFQ-17	1	Rovner (2007)
NEI VFQ-25 adapted (unclear adaptation)	1	Tanveer (2023)
NEI VFQ, unspecified	1	Brody (2005)
Other PROMs		
Impact of Vision Impairment (IVI)	3	Lamoureux (2007); Lek (2017); Kaltenegger (2019)
Night Vision Questionnaire–10 (NVQ-10)	2	Maguire 2004 + Ying 2008 (2004); Lek (2017)
Melbourne Low-Vision Activities of Daily Living Index (MLVAI)	2	Smith (2005); Riazi (2016)
Vision-Specific Sickness Impact Profile (SIPV)	1	Brody (2001)
Vision-Related Quality of Life Core Measure (VCM1)	1	Reeves (2004)
Age-related Macular Degeneration Self-Efficacy Questionnaire (AMD-SEQ)	1	Brody (2005)
Daily Living Tasks Dependent on Vision (DLTV)	1	Stevenson (2005)
Visual Function Index–14 (VF-14)	1	Brunner (2013)
Chinese-version Low Vision Quality of Life questionnaire (CLVQOL)	1	Tao (2016)
Functional Reading Independence Index (FRI)	1	Heier (2020)

PROM = patient-reported outcome measure.

All PROM instrument abbreviations are expanded at first mention in the PROM Name column.

### Risk of Bias Assessment

Risk of Bias 2 assessment focusing on baseline data indicated that 71.1% (27/38) of trials were at a low risk of bias in the randomization process (Domain 1; Table 3). In contrast, 15.8% (6/38) of studies were at a high risk of bias due to missing outcome data (Domain 3, Table 3). All trials were rated at a low risk of bias for outcome measurement at baseline (Domain 4).

**Table 3:** Quality Assessment (Modified Risk of Bias 2)

Author, yr	Domain 1 RoB	Domain 3 RoB	Domain 4 RoB
Brody, 2001	Some concerns	High risk	Low risk
Dong, 2004	Low risk	Low risk	Low risk
Maguire, 2004 + Ying, 2008	Low risk	Low risk	Low risk
Reeves, 2004	Low risk	Low risk	Low risk
Brody, 2005	Low risk	High risk	Low risk
Smith, 2005	Low risk	Low risk	Low risk
Stevenson, 2005	Low risk	Low risk	Low risk
Chang, 2007	Low risk	Low risk	Low risk
Lamoureux, 2007	High risk	Low risk	Low risk
Lüke, 2007	Low risk	Low risk	Low risk
Rovner, 2007	Low risk	Low risk	Low risk
Leys, 2008	Low risk	Low risk	Low risk
Bressler, 2009	Low risk	Low risk	Low risk
Odergren, 2010	Some concerns	Low risk	Low risk
Richer, 2011	Low risk	Low risk	Low risk
Heier, 2012	Low risk	Low risk	Low risk
Piermarocchi, 2012	Low risk	Low risk	Low risk
Brunner, 2013	Some concerns	High risk	Low risk
Mielke, 2013	Some concerns	Low risk	Low risk
Rovner, 2013	Low risk	Low risk	Low risk
Rovner, 2014	Low risk	Low risk	Low risk
Huang, 2015	Some concerns	Low risk	Low risk
Markun, 2015	Low risk	Low risk	Low risk
Riazi, 2016	High risk	High risk	Low risk
Tao, 2016	Some concerns	Low risk	Low risk
Akuffo, 2017	Low risk	Low risk	Low risk
Lek, 2017	Low risk	Low risk	Low risk
Lee, 2018	Low risk	Low risk	Low risk
Kaltenegger, 2019	Some concerns	High risk	Low risk
Heier, 2020	Low risk	High risk	Low risk
Kunimoto, 2020	Low risk	Low risk	Low risk
Visser, 2020_1	Some concerns	Low risk	Low risk
Visser, 2020_2	Low risk	Low risk	Low risk
Gabrielle, 2023	Low risk	Low risk	Low risk
Tanveer, 2023	High risk	Low risk	Low risk
Boyer, 2024	Low risk	Low risk	Low risk
Jackson, 2024	Low risk	Low risk	Low risk
Lanzetta, 2024	Low risk	Low risk	Low risk

RoB = Risk of Bias.

Risk of Bias 2 domains (baseline-only): Domain 1: bias arising from the randomization process; Domain 3: bias due to missing outcome data; Domain 4: bias in measurement of the outcome. For details regarding modifications, see Supplementary Data 2.

### Baseline Functional Burden by Disease Stage

Baseline patient-reported functioning varied markedly by AMD stage and trial entry criteria (Table 4). Participants with early or intermediate AMD entered trials with high functional status, as evidenced by NEI VFQ-25 composite scores ranging from 75.34 to 89.13.^18^^,^^29^ In nAMD trials, baseline scores were substantially lower with greater variance, ranging from 59.1 to 77.9 on the NEI VFQ-25.^26^^,^^32^ The lowest baseline NEI VFQ-25 (standard version) scores occurred in low-vision and rehabilitation cohorts.^50^

**Table 4:** Study Populations and PROM Results

Author, yr	Number of Patients	Female Proportion	Mean Age (SD)	Visual Acuity Metric	Better Eye	Worse Eye	Other Visual Acuity Grouping	PROM Name	Number of Patients with PROM Value	PROM Mean (SD)
Brody, 2001	151	68%	80.11 (6.21)	logMAR: mean (SD)	1.16 (0.46)	1.66 (0.60)		SIPV	151	7.3 (5.96)
								NEI VFQ-39	151	56.98 (13.84)
Dong, 2004	789	53%	Grp 1 (n=454): median 77 Grp 2 (n=335): median 79	ETDRS (Snellen), mean	Grp 1: 20/64 Grp 2: 20/64	Grp 1: 20/320 Grp 2: 20/400		NEI VFQ-39	789	64 (n.r.)
Maguire, 2004 + Ying, 2008	1052	61%	71 (7.6)	ETDRS (Snellen categories): n (%)	20/12: 68 (6.5%) 20/16: 277 (26.3%) 20/20: 336 (31.9%) 20/25: 261 (24.8%) 20/32: 89 (8.5%) 20/40: 21 (2.0%)	20/12: 11 (1.1%) 20/16: 103 (9.8%) 20/20: 248 (23.6%) 20/25: 304 (28.9%) 20/32: 256 (24.3%) 20/40: 130 (12.4%)	All eyes: 20/12: 79 (3.8%) 20/16: 380 (18.1%) 20/20: 584 (27.8%) 20/25: 565 (26.9%) 20/32: 345 (16.4%) 20/40: 151 (7.1%)	NEI VFQ-25	1051	88 (10)
								NVQ-10	1051	70 (20)
Reeves, 2004	226	66%	Grp 1: median 81 (IQR: 77–84) Grp 2: median 80 (IQR: 76–85) Grp 3: median 83 (IQR: 78–86)	logMAR: median (IQR)			Grp 1: 0.81 (0.48–1.00) Grp 2: 0.90 (0.56–1.08) Grp 3: 0.62 (0.44–1.00)	VCM1	226	Grp 1: median 2.1 (IQR: 1.7–2.8) Grp 2: median 2.2 (IQR: 1.5–2.7) Grp 3 median 2.2 (IQR: 1.4–2.7)
Brody, 2005	231	67%	80.89 (6.12)	logMAR: mean (SD)	1.11 (0.42)			NEI VFQ, unspecified	231	59.14 (13.23)
								AMD-SEQ	231	71.34 (15.57)
Smith, 2005	243	65%	Grp 1: median 81 (IQR: 77–85) Grp 2: median 81 (IQR: 77–85) Grp 3: median 81 (IQR: 76–86)	logMAR: median (IQR)	Grp 1: 0.82 (0.62–1.12) Grp 2: 0.92 (0.63–1.19) Grp 3: 1.00 (0.66–1.00)			NEI VFQ-25	229	50 (13.3)
								MLVAI	226	28.4 (4.4)
Stevenson, 2005	199	57%	75.3 (6.4)	logMAR: mean (SD)			Study eye = better eye (n = 117): 0.55 (0.22) Study eye = worse eye (n=82): 0.65 (0.22)	DLTV	199	Dimension 1: 52.7 (4.0) Dimension 2: 80.5 (2.4) Dimension 3: 82.7 (2.1) Dimension 4: 68.3 (3.4)
Chang, 2007	716	65%	77.1 (7.3)	ETDRS (letters): mean (SD)			Study eye: 53.5 (13.3) Fellow eye: 55.3 (28.9)	NEI VFQ-25 + 6 appendix items	716	69.3 (19)
Lamoureux, 2007	56	66%	78.9 (5.7)	logMAR: mean (SD)	–0.25 (0.27)	0.39 (0.24)		IVI	56	2.2 (1.9)
Lüke, 2007	50	54%	77.4 (6.3)	ETDRS (letters): mean (SD)			n.r.: 35.9 (11)	NEI VFQ-39	50	Only subscales reported: General health: 49.4 (17.2) General vision: 46.2 (15.8) Ocular pain: 51.5 (6.4) Near activities: 60.6 (30.5) Distance activities: 70.2 (26.5) Social functioning: 78.4 (24.3) Mental health: 62.1 (25.7) Role difficulties: 62.1 (30.4) Dependency: 75.0 (30.9) Color vision: 93.8 (17.8) Peripheral vision: 76.5 (26.6)
Rovner, 2007	206	70%	81.2 (5.9)	logMAR: mean (SD)			n.r.: 0.60 (0.39)	NEI VFQ-17	206	34.52 (13.46)
Leys, 2008	578	59%	77.1 (7.1)	ETDRS (letters): mean (SD)			Study eye: 52.28 (12.3)	NEI VFQ-25	569	Near vision: 54.7 (24.6) Distance vision: 64.15 (23.5) Role limitations: 63.5 (29.2) Dependency: 74.6 (28.3)
Bressler, 2009	423	50%	77.04 (7.98)	ETDRS (letters): mean (SD)			Study eye: 46.53 (13.1) Fellow eye: 60.42 (28.38)	NEI VFQ-25 + 6 appendix items	418	69.87 (21.1)
Odergren, 2010	98	60%	74.9 (9.3)	ETDRS (letters): mean (SD)			n.r.: 60.2 (10.2)	NEI VFQ-25	98	67.7 (10.9)
Richer, 2011	60	5%	74.9 (10)	ETDRS (letters): mean (SD)			Right eye: 95.2 (8) Left eye: 91.0 (16)	NEI VFQ-25	60	85 (10)
Heier, 2012	2412	57%	76 (8.6)	ETDRS (letters): mean (SD)		53.8 (13.6)		NEI VFQ-25	2403	71.4 (18.1)
Piermarocchi, 2012	145	60%	72.5 (6.98)	ETDRS (letters): mean (SD)			n.r.: 81.86 (5.74)	NEI VFQ-25	145	81.98 (13.48)
Brunner, 2013	49	76%	80.6 (6.3)	logMAR: mean (SD)			n.r.: 0.54 (0.25)	VF-14	49	70.5 (23.9)
Mielke, 2013	20	65%	79 (3.4)	Decimal visual acuity (Snellen decimal): mean (SD)	0.125 (0.08)			NEI VFQ-25	16	All subscales measure but only social functioning reported: 39.3 (23.5)
Rovner, 2013	241	64%	82.8 (6.9)	logMAR: mean (SD)	0.57 (0.29)			NEI VFQ-39	241	66 (14.2)
Rovner, 2014	182	71%	84.1 (6.7)	logMAR: mean (SD)	0.66 (0.37)			NEI VFQ-25‑specific subscales only	188	Social functioning: 65.4 (28.1) Mental health: 46.7 (27.6) Role difficulties: 44.8 (19.1) Dependency: 53.3 (32.3)
Huang, 2015	108	57%	69.12 (7.32)	logMAR: mean (SD)			n.r.: 0.32 (0.21)	NEI VFQ-25	108	75.34 (15.52)
Markun, 2015	169	63%	76.7 (8.2)	ETDRS (letters): median (IQR)			Grp 1: 58 (43–73) Grp 2: 62 (47–73)	NEI VFQ-25	169	
Riazi, 2016	54	57%	69.62 (8.34)	logMAR: mean (SD)			n.r.: 0.53 (0.48)	MLVAI	54	31.81 (5.38)
Tao, 2016	100	46%	71.46 (7.55)	logMAR: mean (SD)			n.r.: 0.65 (0.37)	CLVQOL	100	79.93 (17.28)
Akuffo, 2017	118	66%	64.7 (9.04)	ETDRS (letters): mean (SD)			Study eye: 100.06 (5.72) Fellow eye: 95.3 (11.58)	NEI VFQ-25	118	89.13 (9.63)
Lek, 2017	292	73%	Median 70 (range: 51–89)	ETDRS (letters): mean (SEM)		83 (0.5)		NVQ-10	287	83.4 (SEM: 1.0)
								IVI	284	2.88 (SEM: 0.01)
Kaltenegger, 2019	37	57%	Median 72 (IQR: 67.5–79)	logMAR: median (IQR)	0.7 (0.52–0.8)			IVI	25	median 0.96 (IQR: 0.67–1.34)
Lee, 2018	318	30%	70.6 (8.2)	ETDRS (letters): mean (SD)			n.r.: 58.36 (11.4)	NEI VFQ-25	318	77.18 (13.26)
Heier, 2020	1881	60%	78 (8.1)	ETDRS (letters): mean (SD)			n.r.: 66 (9.8)	NEI VFQ-25 + 6 appendix items	1721	64.63 (16.98)
								FRI	1698	2.68 (0.82)
Kunimoto, 2020	1888	56%	76.2 (8.3)	ETDRS (letters): mean (SD)			Study eye: 56.7 (12.6)	NEI VFQ-25	1888	77.9 (15)
Visser, 2020_1	191	67%	77.3 (6.5)	ETDRS (letters): mean (SD)			n.r.: 64.3 (13.4)	NEI VFQ-39	191	67.6 (19.3)
Visser, 2020_2	185	57%	79.4 (10.2)	ETDRS (letters): mean (SD)	0.30 (0.18)	0.11 (0.14)	Binocular function: 0.30 (0.19)	NEI VFQ-39	185	51.9 (19)
Gabrielle, 2023	89	66%	83.3 (8.2)	ETDRS (letters): mean (SD)			n.r.: 22 (22.5)	NEI VFQ-25	89	59.1 (21.6)
Tanveer, 2023	88	64%	67.28 (8)	logMAR: mean (SD)			n.r. 0.89 (0.08)	NEI VFQ-25 adapted (unclear adaptation)	88	37.4 (4.14)
Boyer, 2024	100	68%	75.35 (7)	ETDRS (letters): mean (SD)			n.r.: 70.49 (4.91)	NEI VFQ-25	100	79.57 (12.73)
Jackson, 2024	411	58%	78 (7.1)	ETDRS (letters): mean (SD)			n.r.: 68.63 (13.17)	NEI VFQ-25	Grp 1: 274 Grp 2: 137	Grp 1: median 87 (IQR: 77–94) Grp 2: median 87 (IQR: 70–93)
Lanzetta, 2024	1009	55%	74.5 (8.4)	ETDRS (letters): mean (SD)			n.r.: 59.6 (13.3)	NEI VFQ-25	954	77.27 (14.98)

AMD-SEQ = Macular Degeneration Self-Efficacy Questionnaire; CLVQOL = Chinese-version Low Vision Quality of Life questionnaire; DLTV = Daily Living Tasks Dependent on Vision; FRI = Functional Reading Independence Index; Grp = group; IQR = interquartile range; IVI = Impact of Vision Impairment; logMAR = logarithm of the minimum angle of resolution; MLVAI = Melbourne Low-Vision Activities of Daily Living Index; NEI VFQ = National Eye Institute Visual Function Questionnaire; n.r. = not reported; NVQ-10 = Night Vision Questionnaire–10; PROM = patient-reported outcome measure; SD = standard deviation; SEM = standard error of the mean; SIPV = Vision-Specific Sickness Impact Profile; VCM1 = Vision-Related Quality of Life Core Measure; VF-14 = Visual Function Index–14.

### Subscale and Domain Reporting

Subscale-level and domain-level baseline reporting was available for only a minority of trials. Where reported, this was most commonly within the NEI VFQ family, but subscale coverage varied, with some studies omitting selected domains (e.g., driving and general health) or reporting only a limited set of activity subscales. One trial reported baseline subscale means without accompanying measures of dispersion (i.e., standard deviations). Given the use of different NEI VFQ versions and heterogeneous subscale selection and reporting formats, subscale-level analyses could not be synthesized quantitatively across trials.

### PROM Results Stratified by Geographic Location and Ethnicity

Although included RCTs recruited participants across multiple regions, including North America, Europe, Asia, and Oceania, baseline PROM results were not reported stratified by ethnicity, geographic region, language, country, or economic setting. Several multinational trials reported pooled PROM results, and single-country trials differed substantially in AMD phenotype, disease stage, intervention type, trial era, PROM instrument, and eligibility criteria. We therefore did not perform comparisons by region or by high-income versus lower-resource settings, because such comparisons would have been ecological and highly confounded. Consequently, regional variation in baseline patient-reported burden or in PROM reporting completeness could not be reliably assessed.

## Discussion

### Main Findings

This systematic review of vision-related PROMs in AMD RCTs found that incomplete baseline reporting and inconsistent operationalization substantially limited interpretability, cross-trial comparability, and secondary synthesis. A major challenge identified during full-text screening was incomplete reporting of PROM data. Baseline PROM data were frequently unavailable for extraction despite PROM collection being listed among trial outcomes. The absence of baseline PROM distributions and subscale results weakens interpretation of longitudinal PROM change, undermines cross-trial comparability, and limits secondary synthesis. It also hampers assessment of reporting bias because trials often do not clarify whether PROMs were administered and analyzed for all randomized participants or only a subset, nor the extent and handling of missing baseline data.

Among trials with extractable baseline PROM data, the NEI VFQ family predominated. However, operationalization varied considerably across studies, including the use of multiple NEI VFQ versions, occasional incorporation of appendix items, and sporadic reporting of adaptations without sufficient methodological detail to assess comparability. Domain and subscale results were reported less consistently than composite scores, and selective reporting of specific subscales was observed. Beyond the NEI VFQ family, alternative vision-related PROMs were employed only sporadically. The Impact of Vision Impairment questionnaire was the most frequently used alternative, whereas night-vision and low-luminance‑specific measures were rare, thereby limiting the capacity for evidence comparison across these constructs.

Interpretation was further constrained by mismatches between (1) the level of intervention and outcome analysis in most AMD RCTs (typically a single treated study eye) and (2) the level at which vision-related PROMs capture experience, which reflects person-level functioning often encompassing binocular vision. Reporting of treated-eye designation, fellow-eye status, and operational definitions of BSE and WSE classification was insufficient to contextualize PROM findings within binocular function. Because baseline PROM results were not reported by region, ethnicity, language, or economic setting, we could not assess whether PROM burden or reporting completeness varied geographically. We also could not determine whether incomplete baseline PROM reporting reflected absence of data collection, selective nonreporting, incomplete analysis, or lower prioritization of PROM outcomes.

### Results in the Context of the Existing Literature

These findings align with prior observations regarding the inconsistent availability of PROM data in AMD RCTs and the predominant use of NEI VFQ instruments when PROMs are included.^14^ Extending Krezel et al’s observation that PROMs are infrequently incorporated in RCTs of AMD, the present analysis demonstrates that their interpretive utility remains constrained, even when employed, by marked heterogeneity in instrument versions and operationalization, selective reporting of domains and subscales, and heterogeneous documentation of laterality, BSE or WSE status, or binocular visual status, impeding cross-trial comparability and person-level inference.

Uniform PROM application across heterogeneous AMD populations presents ongoing challenges. In early and intermediate AMD, patient-perceived functional limitations may be driven by low-luminance performance, contrast-dependent tasks, and dark adaptation despite relatively preserved BCVA.^2^, ^3^, ^4^ In such contexts, broad questionnaires may co-occur with limited discriminative performance at the upper end of functioning and seem susceptible to ceiling effects. Stage-appropriate PROM selection, with prespecified hypotheses targeting relevant domains, is particularly important for trials addressing earlier disease stages or interventions expected to influence low-luminance function more substantially than high-contrast acuity.^2^

Geographic and resource-setting differences may also influence both baseline patient-reported burden and the feasibility of PROM implementation. In lower-resource eye-care settings, later presentation may result in more advanced AMD at first assessment, which could affect baseline PROM distributions and the domains most relevant to patients. For example, a national study from Bhutan reported that 44.9% of new AMD patients presented with late-stage AMD and that approximately half of nAMD cases had disciform scarring not amenable to treatment at first presentation.^58^ However, our review could not determine whether PROM scores or PROM reporting completeness varied by region, language, ethnicity, or economic setting. Multinational RCTs generally reported pooled PROM results, and single-country trials differed substantially in AMD phenotype, disease stage, intervention type, trial era, PROM instrument, and eligibility criteria. Consequently, comparisons by region or resource setting would have been ecological and highly confounded. Future AMD trials should therefore report baseline PROM distributions alongside AMD stage, visual-function strata, language, geography, ethnicity, and other relevant participant characteristics where feasible and prespecified.

Laterality and binocular vision introduce additional complexity. Vision-related quality of life reflects integrated experience across both eyes (binocular function), and the relationship between monocular acuity and patient-reported functioning is associated with binocular summation and inhibition as well as task demands.^59^^,^^60^ Observational evidence indicates that both BSE and WSE vision contribute meaningfully to vision-related quality of life, with the WSE contribution demonstrating clinical significance.^6^^,^^59^^,^^60^ Subgroup analyses from landmark trials (Minimally Classic/Occult Trial of the Anti-VEGF Antibody Ranibizumab in the Treatment of Neovascular Age-Related Macular Degeneration and Anti-VEGF Antibody for the Treatment of Predominantly Classic Choroidal Neovascularization in Age-Related Macular Degeneration) reported patient-reported gains regardless of whether treatment was delivered to the BSE or WSE, with larger gains more commonly observed after BSE treatment.^13^ Accordingly, trial reports should explicitly specify treated-eye designation, fellow-eye status, and the operational definition employed for BSE and WSE classification when presenting PROM outcomes.

### Limitations

Our findings must be interpreted in light of several limitations. First, our eligibility criteria required the availability of extractable baseline PROM data. This likely introduced a selection toward best-reported trials and excluded major AMD RCTs in which PROMs were collected only after randomization or reported only as follow-up values or change scores. However, this criterion was necessary for the review question, as postrandomization PROM values cannot be assumed to represent patient-reported functional status at trial entry. While many identified RCTs used PROMs as secondary or exploratory endpoints, a significant proportion failed to report baseline values in the primary or secondary publications, often only presenting longitudinal change scores or *P* values. Consequently, our results may reflect the functional burden of patients in trials with high reporting standards rather than providing a truly representative cross-section of the broader AMD clinical trial landscape. Furthermore, while we used a modified Risk of Bias 2 framework to assess baseline data integrity, we did not numerically track the exact number of studies excluded solely due to the absence of extractable baseline PROM data versus those that did not use PROMs at all. Finally, we did not retain a study-level list of full-text articles excluded after detailed review with individual reasons for exclusion. Instead, exclusions were summarized by grouped categories.

### Implications for Research

Future AMD RCTs should select PROMs that align with the target population and the functional domains most likely to change. For intermediate or earlier AMD cohorts with preserved acuity, instruments capturing low-luminance, contrast-related, and activity-specific difficulties may be needed to avoid ceiling effects and detect clinically meaningful change. For nAMD and other settings with larger expected acuity changes, established instruments such as the NEI VFQ remain appropriate, particularly when domain-level hypotheses are prespecified and domain-level results are reported alongside composite scores.

To enable cross-trial comparability and evidence synthesis, trial reports should clearly document instrument name and exact version, language, any modifications or appended items, scoring methods, assessment timing, and prespecified approaches to missing PROM data. Baseline PROM distributions should be routinely presented alongside the number of participants assessed, even when PROMs are secondary outcomes, as baseline values are essential for interpreting change and conducting secondary analyses. Where feasible and prespecified, baseline and follow-up PROM reporting should be linked to AMD stage, visual-function strata, treated-eye status, geography, ethnicity, language, and resource-relevant context to permit evaluation of differential patient-reported burden and improve cross-trial comparability.

To make these recommendations operational, we propose an AMD-specific minimum reporting set for vision-related PROMs in RCTs (Table 5). This proposed set is intended to complement, rather than replace, established patient-reported outcomes guidance such as Consolidated Standards of Reporting Trials extension for patient-reported outcomes.^61^ It is not a validated consensus reporting guideline and does not prescribe which PROM instrument should be used. Instead, it identifies AMD-specific contextual information needed to interpret and compare PROM findings, particularly when unilateral ocular interventions are evaluated using person-level outcomes. Key items include the rationale and status of the PROM in the trial, instrument version and measurement properties, administration procedures, denominators and missing-data handling, AMD phenotype and visual-function context, treated-eye and fellow-eye status, BSE and WSE definitions, binocular visual function where collected, and reporting of baseline and follow-up PROM distributions.

**Table 5:** Reporting Considerations for Age-Related Macular Degeneration Randomized Controlled Trials

Section	Item	Minimum Reporting Item	Details and Examples
A. PROM identification and administration	1	Instrument name and exact version	Full name, abbreviation (e.g., NEI VFQ-25), version/revision, and year.
A. PROM identification and administration	2	Language and cultural adaptation	Language(s) used; whether a validated translation or cultural adaptation.
A. PROM identification and administration	3	Mode of administration	Self-completed vs. interviewer-administered; paper vs. electronic; clinic vs. remote.
A. PROM identification and administration	4	Modifications relative to the original instrument	Any item deletions, additions, rewording, reordering, or shortened forms; rationale.
A. PROM identification and administration	5	Recall period and assessment context	Stated recall period (e.g., “past 4 weeks”) and how assessments are timed relative to key trial visits (e.g., after loading phase, at the end of maintenance).
B. Scoring, missing data, and responder definition	6	Scoring algorithm	Which subscales and composite scores are used; item weighting; transformations (e.g., to 0–100 scale); direction of better scores (higher = better or worse).
B. Scoring, missing data, and responder definition	7	Handling of missing items	Rules for the maximum number and proportion of missing items per scale; imputation methods, if any; handling of “not applicable” responses.
B. Scoring, missing data, and responder definition	8	Responder thresholds and clinically important differences	Prespecified thresholds for meaningful change (if used) and their source (e.g., published MCID, distribution-based).
C. Eye laterality and binocular context	9	Study eye (treated eye) definition	Criteria used to select the treated eye; how ties and conflicts were managed; whether PROM analyses are anchored to the study eye or person-level.
C. Eye laterality and binocular context	10	Fellow-eye status at baseline	At minimum, baseline fellow-eye VA; report whether substantial changes in fellow-eye status occurred during follow-up (e.g., conversion, new treatment).
C. Eye laterality and binocular context	11	Better-seeing/worse-seeing eye definition	Operational definition (e.g., by VA in ETDRS letters); timepoint used (baseline vs. time-varying); whether reclassification over time was allowed.
C. Eye laterality and binocular context	12	Binocular visual function measures	Reporting of binocular distance VA as a minimum; where available, near VA or reading measures, contrast sensitivity, or stereopsis, or a stated rationale if no binocular measure was obtained.
D. PROM timing and statistical reporting	13	Assessment schedule	All PROM timepoints; alignment with primary anatomical and VA endpoints; whether PROMs were prespecified as primary and secondary outcomes.
D. PROM timing and statistical reporting	14	Descriptive distributions at baseline and follow-up	For each key PROM scale: central tendency and spread (mean ± SD and/or median [IQR]); indicate potential floor or ceiling effects if present.
D. PROM timing and statistical reporting	15	Subscale versus composite reporting	Which subscales are reported and how multiplicity is handled (e.g., adjustment or prespecified hierarchy); whether only a composite score is used despite a multidomain instrument.

IQR = interquartile range; MCID = minimal clinically important difference; NEI VFQ = National Eye Institute Visual Function Questionnaire; PROM = patient-reported outcome measure; SD = standard deviation; VA = visual acuity.

For any age-related macular degeneration randomized controlled trials reporting vision-related patient-reported outcomes (PROM), the minimum information should be provided to support interpretation, comparison, and synthesis.

Existing PROM reporting guidance provides general recommendations for instrument selection and reporting. However, AMD trials pose additional interpretive challenges because interventions are typically unilateral while PROMs reflect person-level (often binocular) functioning. Accordingly, AMD RCT reports should explicitly specify treated-eye designation, fellow-eye status, and binocular context when presenting PROM outcomes.

## Conclusion

Across AMD RCTs, the interpretive value of vision-related PROMs is constrained by incomplete baseline reporting, heterogeneity in NEI VFQ versions and operationalization, limited reporting of domains and subscales, and insufficient documentation of treated-eye and fellow-eye status. Enhanced consistency in stage-appropriate PROM selection and reporting, coupled with routine documentation of laterality and binocular context, would substantially improve interpretability and increase the potential for evidence synthesis and patient-centered evaluation of interventions.

## Declaration of Generative AI and AI-Assisted Technologies in the Writing Process

During the preparation of this work the authors used ChatGPT (OpenAI) for language editing and spelling checks. After using this tool, the authors reviewed and edited the content as needed and take full responsibility for the content of the publication.
