# Supplementary material for: Vision-Related Patient-Reported Outcomes in Randomized Controlled Trials of Age-Related Macular Degeneration: A Systematic Review
Source: Ophthalmol Sci. 2026 Jun 30;6(9):101308. doi: 10.1016/j.xops.2026.101308 (PMC13449777; doi:10.1016/j.xops.2026.101308)
Supplement: Supplementary file 1 [file mmc1.pdf]

SUPPLEMENTARY MATERIAL

Supplementary Data 1 – Search Strategy

Search results for ***Patient-reported outcomes (PRO) in age-related macular degeneration (AMD) across disease stages, ethnicity and geography***

Date: September 27th, 2023

Search Protocols, Printscreens and Reference Files:

- DOKU_Embase.alisbe.pdf
- DOKU_Medline Ovid_alisbe.pdf
- DOKU_Web of Science_alisbe.pdf
- DOKU_Scopus_alisbe.pdf
- DOKU_DB-Search-Histories_alisbe.xlsx
- PRO Measures for AMD.txt
- PRO Measures for AMD.enlx

	**Deduplication**	
	Before	after
Medline	1722	1718
EMBASE	2493	1463
Web of Science	1534	551
Scopus	1607	309
Pool	7356	**4041***

* May still contain duplicates, if available adapt PRISMA flowchart accordingly

Reference files: PRO Measures for AMD.enlx (compressed EN Library file)
PRO Measures for AMD.txt (RIS-file)

Before:

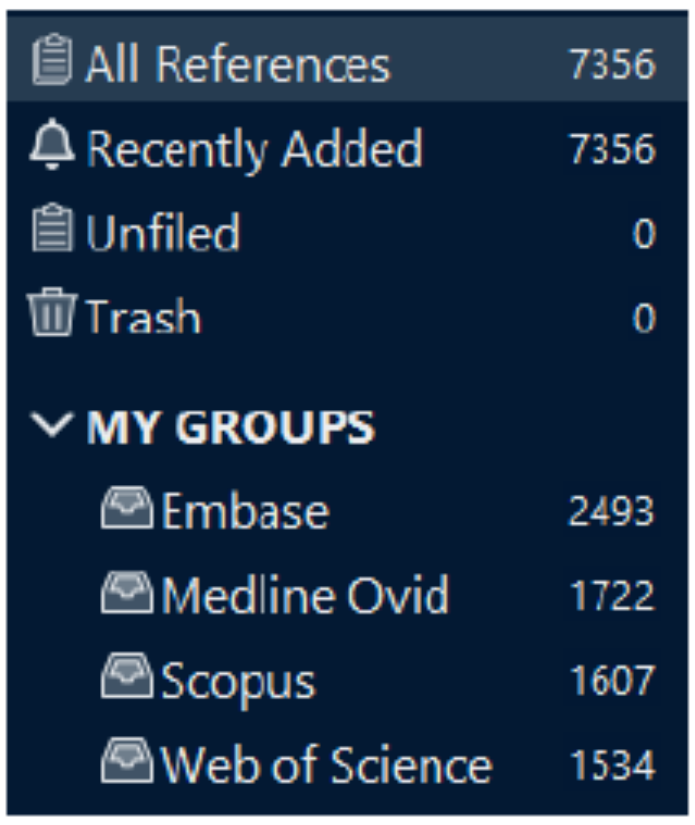

after:

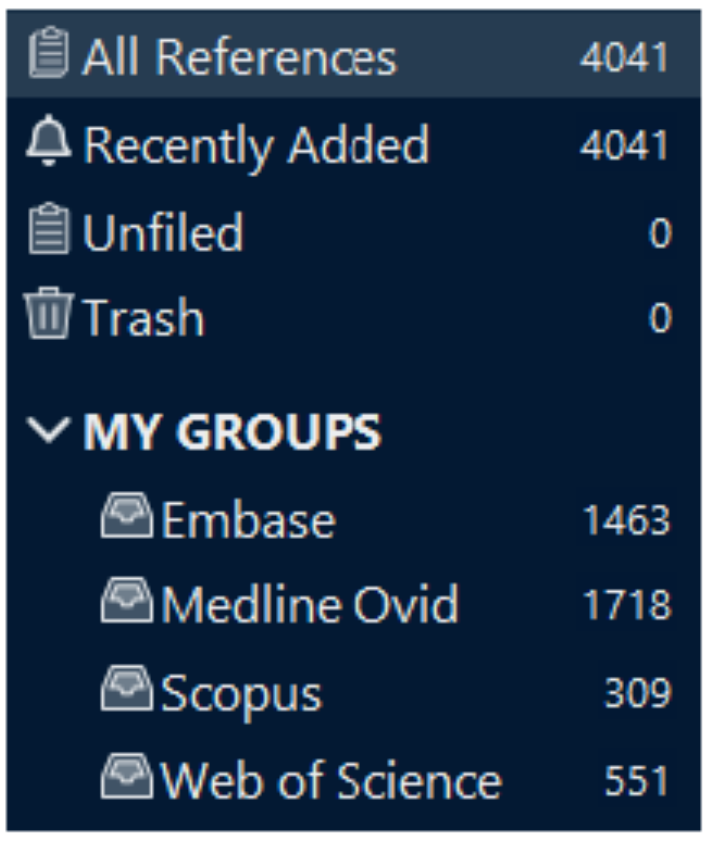

Medline

Database(s): **Ovid MEDLINE(R) and Epub Ahead of Print, In-Process, In-Data-Review & Other Non-Indexed Citations and Daily** 1946 to September 25, 2023
Search Strategy:

#	Searches	Results
1	exp Macular Degeneration/ or exp Retinal Drusen/ or exp Choroidal Neovascularization/ or (macula* adj2 (disease or degen* or lutea or atroph*)).ti,ab. or (AMD or ARMD or nAMD or wAMD or maculopath* or "polypoidal choroidal vasculopathy" or PCV or "geographic atrophy" or "subretinal drusenoid deposit*" or SDD or "pseudoretincular drusen" or "aneurysmal type 1").ti,ab.	60844
2	Patient Reported Outcome Measures/ or ((Vision Disorders/ or Vision, Low/ or exp Visual Acuity/ or exp Vision, Ocular/) and (exp "Surveys and Questionnaires"/ or exp "Quality of Life"/ or exp Health Status/)) or ((vision or ophthal* or visual or sight* or macula*) adj6 (HR-PRO or HRPRO or HRQL or HRQoL or QL or QoL)).ti,ab. or ((vision or ophthal* or visual or sight* or macula*) adj6 ("quality of life" or "health index*" or "health indices" or "health profile*" or "health status")).ti,ab. or ((patient or self or child or parent or carer or proxy) adj1 (appraisal* or appraised or report or reported or reporting or rated or rating* or based or assessed or assessment*) adj6 (vision or ophthal* or visual or sight* or macula*)).ti,ab. or ((impair* or disability or loss or low or acuity or function or functional or functions or activit* or task* or subjective or utility or utilities or wellbeing or "well being" or burden) adj2 (index or indices or instrument or instruments or measure or measures or questionnaire* or profile or profiles or scale or scales or score or scores or status or survey or surveys) adj6 (vision or ophthal* or visual or sight* or macula*)).ti,ab.	38115
3	1 and 2	1749
4	3 not (animals not humans).sh.	1748
5	limit 4 to (congress or editorial or letter or preprint)	26
6	4 not 5	1722

1. Diagnostic accuracy of self-reported age-related macular degeneration in the ASPREE Longitudinal Study of Older Persons.

   McGuinness MB, Robman L, Hodgson LAB, Tran C, Woods RL, Owen AJ, McNeil JJ, Makeyeva G, Abhayaratna WP, Guymer RH

   Eye. 2023 Sep 20.

   [Journal Article]

   UI: 37731049

   Digital Object Identifier
   https://dx.doi.org/10.1038/s41433-023-02754-y

   Authors Full Name
   McGuinness, Myra B, Robman, Liubov, Hodgson, Lauren A B, Tran, Cammie, Woods, Robyn L, Owen, Alice J, McNeil, John J, Makeyeva, Galina, Abhayaratna, Walter P, Guymer, Robyn H

EMBASE

Embase session results (27 Sep 2023)

No.	Query	Results
#5	#4 NOT ([conference abstract]/lim OR [editorial]/lim OR [letter]/lim OR [note]/lim OR [preprint]/lim)	2493
#4	#3 NOT (('animal'/de OR 'animal experiment'/exp OR 'nonhuman'/de) NOT ('human'/exp OR 'human experiment'/de))	3303
#3	#1 AND #2	3315
#2	'patient-reported outcome'/de OR (('visual impairment'/de OR 'low vision'/de OR 'vision'/exp OR 'visual acuity'/exp) AND ('questionnaire'/exp OR 'quality of life'/exp OR 'quality of life assessment'/de OR 'wellbeing'/exp OR 'health status'/exp)) OR (((vision OR ophthal* OR visual OR sight* OR macula*) NEAR/6 ('hr pro' OR hrpro OR hrql OR hrqol OR ql OR qol)):ti,ab,kw) OR (((vision OR ophthal* OR visual OR sight* OR macula*) NEAR/6 ('quality of life' OR 'health index*' OR 'health indices' OR 'health profile*' OR 'health status')):ti,ab,kw) OR (((patient OR self OR child OR parent OR carer OR proxy) NEXT/1 (appraisal* OR appraised OR report OR reported OR reporting OR rated OR rating* OR based OR assessed OR assessment*) NEAR/6 (vision OR ophthal* OR visual OR sight* OR macula*)):ti,ab,kw) OR (((impair* OR disability OR loss OR low OR acuity OR function OR functional OR functions OR activit* OR task* OR subjective OR utility OR utilities OR wellbeing OR 'well being' OR burden) NEAR/2 (index OR indices OR instrument OR instruments OR measure OR measures OR questionnaire* OR profile OR profiles OR scale OR scales OR score OR scores OR status OR survey OR surveys) NEAR/6 (vision OR ophthal* OR visual OR sight* OR macula*)):ti,ab,kw)	94850
#1	'macular degeneration'/exp OR 'subretinal drusenoid deposit'/exp OR 'subretinal neovascularization'/exp OR	83293

No.	Query	Results
	((macula* NEAR/2 (disease OR degen* OR lutea OR atroph*)):ti,ab,kw) OR amd:ti,ab,kw OR armd:ti,ab,kw OR namd:ti,ab,kw OR wamd:ti,ab,kw OR maculopath*:ti,ab,kw OR 'polypoidal choroidal vasculopathy':ti,ab,kw OR pcv:ti,ab,kw OR 'geographic atrophy':ti,ab,kw OR 'subretinal drusenoid deposit*':ti,ab,kw OR sdd:ti,ab,kw OR 'pseudoretincular drusen':ti,ab,kw OR 'aneurysmal type 1':ti,ab,kw	

Web of Science

Web of Science Search Strategy (v0.1)

Database: Web of Science Core Collection

Entitlements:

- WOS.IC: 1993 to 2023
- WOS.CCR: 1985 to 2023
- WOS.SCI: 1900 to 2023
- WOS.AHCI: 1975 to 2023
- WOS.BHCI: 2005 to 2023
- WOS.BSCI: 2005 to 2023
- WOS.ESCI: 2018 to 2023
- WOS.ISTP: 1990 to 2023
- WOS.SSCI: 1900 to 2023
- WOS.ISSHP: 1990 to 2023

Searches:

1: TS=((macula* NEAR/2 (disease OR degen* OR lutea OR atroph*)) OR AMD OR ARMD OR nAMD OR wAMD OR maculopath* OR "polypoidal choroidal vasculopathy" OR PCV OR "geographic atrophy" OR "subretinal drusenoid deposit*" OR SDD OR "pseudoretincular drusen" OR "aneurysmal type 1") Date Run: Wed Sep 27 2023 15:55:44 GMT+0200 (Mitteleuropäische Sommerzeit) Results: 70479

2: TS=(((vision OR ophthal* OR visual OR sight* OR macula*) NEAR/6 (HR-PRO or HRPRO or HRQL or HRQoL or QL or QoL)) OR ((vision OR ophthal* OR visual OR sight* OR macula*) NEAR/6 ("quality of life" or "health index*" or "health indices" or "health profile*" OR "health status")) OR ((vision OR ophthal* OR visual OR sight* OR macula*) NEAR/6 ((patient or self or child or parent or carer or proxy) NEAR/0 (appraisal* or appraised or report or reported or reporting or rated or rating* or based or assessed or assessment*))) OR ((vision OR ophthal* OR visual OR sight* OR macula*) NEAR/6 ((impair* or disability or loss or low or acuity or function or functional or functions or activit* or task* or subjective or utility or utilities or wellbeing or "well being" or burden) NEAR/2 (index or indices or instrument or instruments or measure or measures or questionnaire* or profile or profiles or scale or scales or score or scores or status or survey or surveys)))) Date Run: Wed Sep 27 2023 16:04:46 GMT+0200 (Mitteleuropäische Sommerzeit) Results: 27830

3: #1 AND #2 Date Run: Wed Sep 27 2023 16:05:08 GMT+0200 (Mitteleuropäische Sommerzeit) Results: 1639

4: #1 AND #2 and Meeting Abstract or Editorial Material or Letter (Exclude – Document Types) Date Run: Wed Sep 27 2023 16:05:37 GMT+0200 (Mitteleuropäische Sommerzeit) Results: 1534

Scopus

Advanced search

< Basic Search | Advanced | Search tips ⓘ

Enter query string

Outline query | Add Author name / Affiliation | Search

ALL("Cognitive architectures") AND AUTHOR-NAME(smith)
TITLE-ABS-KEY(*somatic complaint wom?n) AND PUBYEAR AFT 1993
SRCTITLE(*field ornith*) AND VOLUME(75) AND ISSUE(1) AND PAGES(53-66)

Operators

- AND +
- OR +
- AND NOT +
- PRE/ +
- W/ +

Field codes ⓘ

- Textual Content
- Affiliations
- Authors
- Biological Entities
- Chemical Entities
- Conferences
- Document
- Editors
- Funding

2 TITLE-ABS-KEY (((vision OR ophthal* OR visual OR sight* OR macula*) W/6 (hr-pro OR hrpro OR hrql OR hrqol OR ql OR qol)) OR ((vision OR ophthal* OR visual OR sight* OR macula*) W/6 ("quality of life" OR "health index*" OR "health indices" OR "health profile*" OR "health status")) OR ((vision OR ophthal* OR visual OR sight* OR macula*) W/6 ((patient OR self OR child OR parent OR carer OR proxy) W/0 View More — 30,028 document results

1 TITLE-ABS-KEY ((macula* W/2 (disease OR degen* OR lutea OR atroph*)) OR amd OR armd OR namd OR wamd OR maculopath* OR "polypoidal choroidal vasculopathy" OR pcv OR "geographic atrophy" OR "subretinal drusenoid deposit*" OR sdd OR "pseudoretincular drusen" OR "aneurysmal type 1") — 101,962 document results

Search history

Combine queries... *e.g. #1 AND NOT #3*

4 (TITLE-ABS-KEY ((macula* W/2 (disease OR degen* OR lutea OR atroph*)) OR amd OR armd OR namd OR wamd OR maculopath* OR "polypoidal choroidal vasculopathy" OR pcv OR "geographic atrophy" OR "subretinal drusenoid deposit*" OR sdd OR "pseudoretincular drusen" OR "aneurysmal type 1")) AND (TITLE-ABS-KEY (((vision OR ophthal* OR visual OR sight* OR macula*) W/6 (hr-pro OR hrpro OR hrql OR hrqol OR ql OR qol)) OR ((vision OR ophthal* OR visual OR sight* OR macula*) W/6 ("quality of life" OR "health index*" OR "health indices" OR "health profile*" OR "health status")) OR ((vision OR ophthal* OR visual OR sight* OR macula*) W/6 ((patient OR self OR child OR parent OR carer OR proxy) W/0 (appraisal* OR appraised OR report OR reported OR reporting OR rated OR rating* OR based OR assessed OR assessment*))) OR ((vision OR ophthal* OR visual OR sight* OR macula*) W/6 ((impair* OR disability OR loss OR low OR acuity OR function OR functional OR functions OR activit* OR task* OR subjective OR utility OR utilities OR wellbeing OR "well being" OR burden) W/2 (index OR indices OR instrument OR instruments OR measure OR measures OR questionnaire* OR profile OR profiles OR scale OR scales OR score OR scores OR status OR survey OR surveys))))) AND (EXCLUDE (DOCTYPE , "le") OR EXCLUDE (DOCTYPE , "no") OR EXCLUDE (DOCTYPE , "ed")) View Less — 1,608 document results

3 (TITLE-ABS-KEY ((macula* W/2 (disease OR degen* OR lutea OR atroph*)) OR amd OR armd OR namd OR wamd OR maculopath* OR "polypoidal choroidal vasculopathy" OR pcv OR "geographic atrophy" OR "subretinal drusenoid deposit*" OR sdd OR "pseudoretincular drusen" OR "aneurysmal type 1")) AND (TITLE-ABS-KEY (((vision OR ophthal* OR visual OR sight* OR macula*) W/6 (hr-pro OR hrpro OR hrql View More — 1,632 document results

Supplementary Data 2 - Detailed Eligibility Criteria

- Study Design: Randomized Controlled Trials (RCTs)* reporting original patient data.
- Population: Participants diagnosed with Age-Related Macular Degeneration (AMD).
- Sample Size: A minimum of 20 participants randomized
- Outcomes:
    - Use of a vision-related Patient-Reported Outcome Measure (PROM).
    - Availability of baseline data for both Visual Acuity (VA) and the vision-related PROM for AMD patients
- Data Sources of Information:
    - Peer-reviewed publications
    - Supplementary Material
    - FDA/other supporting documents
    - Trial registries (e.g., ClinicalTrials.gov)
- Restrictions: No restrictions were imposed regarding publication language or methodological quality.
- Screening/Search:
    - Title/Abstract Phase: To minimize the risk of excluding relevant studies, a low threshold was applied during the title and abstract screening: records were included if they contained any indication of a PROM. The determination of whether the PROM was specifically vision-related was reserved for the full-text screening stage.
    - Additional study identification: We reviewed the bibliographies of full-text articles to identify citations of other trials that may have evaluated vision-related PROMs. Additionally, content experts were consulted to identify potentially missed trials. All resulting suggestions were formally screened against the inclusion criteria.

*Protocol Deviation: The original protocol intended to include observational studies. However, the lack of standardized indexing for these study designs hindered their exhaustive retrieval. This limitation posed a high risk of missing relevant studies, which would have resulted in a distorted representation of the available

evidence. Therefore, to maintain feasibility and ensure a robust dataset, the review was restricted to Randomized Controlled Trials (RCTs).

Supplementary Data 3 – Risk of bias (RoB 2) approach and modifications

Risk of bias was assessed using the Cochrane Risk of Bias 2 (RoB 2) tool, but in a reduced form tailored to baseline data (i.e., outcomes measured at baseline / pre-intervention). Because our focus was on baseline values only, signalling questions and domains that relate to post-randomization processes (e.g., deviations from intended interventions, follow-up, outcome measurement after allocation, selective reporting) were not applicable and were therefore not assessed.

RoB 2 domains assessed (reduced set)

We retained signalling questions from the following RoB 2 domains:

Domain 1: Bias arising from the randomization process

- *1.1 Was the allocation sequence random?*
- *1.2 Was the allocation sequence concealed until participants were enrolled and assigned to interventions?*
- *1.3 Did baseline differences between intervention groups suggest a problem with the randomization process?*

Domain 3: Bias due to missing outcome data

- *3.1 Were data for this outcome available for all, or nearly all, participants randomized?*
- *3.2 If not: Is there evidence that the result was not biased by missing outcome data?*
- *3.3 If needed: Could missingness in the outcome depend on its true value?*
- *3.4 If needed: Is it likely that missingness* in the outcome depended on its true value?

Domain 4: Bias in measurement of the outcome

- *4.1 Was the method of measuring the outcome appropriate?*
- *4.2 Could measurement or ascertainment of the outcome have differed between intervention groups?*
