# Supplementary material for: Vision-Related Patient-Reported Outcomes in Randomized Controlled Trials of Age-Related Macular Degeneration: A Systematic Review
Source: Ophthalmol Sci. 2026 Jun 30;6(9):101308. doi: 10.1016/j.xops.2026.101308 (PMC13449777; doi:10.1016/j.xops.2026.101308)
Supplement: Supplementary file 2 [file mmc2.pdf]

Supplementary Table 1 - Inclusion and Exclusion Criteria of Included Studies (Ad Verbatim)

Author, Year	Inclusion Criteria	Exclusion Criteria
Brody, 2001	Individuals were recruited who met the following inclusion and exclusion criteria: (1) diagnosis by an ophthalmologist of AMD confirmed by fundus photographs; (2) visual acuity of 20/60 or worse in the better eye and 20/100 or worse in their other eye with habitual correction (i.e., current glasses); (3) no other unstable eye disease or vision loss caused by another eye disease; (4) age 60 or older; (5) adequate hearing, with a hearing aid if necessary, to complete the interview and to respond in normal conversation; (6) physical ability to come to an interview if wheelchair access transportation were provided; (7) no cognitive impairment as assessed by the Orientation-Memory Concentration Test,19 and (8) no current alcohol abuse as assessed by the Short Michigan Alcoholism Screening Test.20	
Dong, 2004	Group N (New CNV)<br>Age: ≥ 50 years<br>CNV cause: AMD<br>Classic CNV: Required<br>Occult CNV: Optional<br>Foveal center: CNV under center of foveal avascular zone<br>Lesion size: ≤ 9 disc areas (if > 3.5 disc areas [8.9 mm²], boundaries must be poorly demarcated)<br>Area of blood: ≤ 50% of lesion<br>Prior laser: Not allowed<br>Best VA, study eye: ≥ 20/100 (ETDRS after refraction)<br>Worst VA, study eye: ≤ 20/800 (ETDRS after refraction)<br>Fellow eye VA: ≥ 20/800	Group N: Ocular – Any previous laser or photodynamic therapy involving the foveal center<br>– Media opacity precluding clear fundus photography<br>– Other ocular disease that could confound vision (e.g., advanced glaucoma, significant cataract, diabetic retinopathy)<br>– CNV from non-AMD causes<br>Systemic – Serious medical illness limiting survival or follow-up<br>– Inability to undergo surgery or anesthesia if randomized to surgery<br>Other – Unwillingness or inability to adhere to protocol visits<br>– Recent participation in another interventional trial Group B: Age ≥ 50 years

	Consent / follow-up: Signed informed consent and willingness for long-term follow-up.<br><br>Group B (Predominantly hemorrhagic CNV)<br>Age: ≥ 50 years<br>CNV cause: AMD with substantial subretinal or sub-RPE hemorrhage<br>Classic CNV: Optional<br>Occult CNV: Optional<br>Foveal center: Blood or CNV under center of foveal avascular zone<br>Lesion size: ≤ 9 disc areas (≈ 22.9 mm²); no upper limit except ≥ 75% of the blood must be posterior to the equator<br>Area of blood: ≥ 50% of lesion<br>Prior laser: Optional, cannot involve foveal center<br>Best VA, study eye: ≥ 20/100 (ETDRS after refraction)<br>Worst VA, study eye: Light perception (typically ≤ 20/800)<br>Fellow eye VA: ≥ 20/800<br>Consent / follow-up: Written informed consent and willingness to complete protocol follow-up.	Diagnosis CNV secondary to AMD with substantial subretinal or sub-RPE hemorrhage<br>Lesion characteristics • Blood occupying ≥ 50 % of the total lesion area<br>Total lesion size ≤ 9 disc areas (≈ 22.9 mm²)<br>Foveal involvement Blood or CNV extends under the center of the fovea<br>Prior treatment Prior laser photocoagulation permitted only if it did not involve the foveal center<br>Visual acuity (study eye) Between 20/100 and light perception (typically ≤ 20/800)<br>Fellow eye VA ≥ 20/800<br>Consent Written informed consent and willingness to complete protocol follow-up
Maguire 2004 + Ying 2008, 2004	Patient inclusion criteria: Age =>50 years, Signed informed consent form, Available for 5 years of follow-up<br><br>Eligibility criteria for each eye:<br>10+ large drusen (> 125 microns) within 3000 microns of the foveal center, Visual acuity of 20/40 or better (ETDRS equivalent), No evidence of exudative AMD, No serous PED of any size, No geographic atrophy within 500 microns of the foveal center, Total area of geographic atrophy within 3000 microns of the foveal center	History of ocular laser or pharmacologic therapy

	must be µ1 MPS Disc Area, No macular edema or signs of diabetic retinopathy more severe than 10 red dots (microaneurysms or blot hemorrhages), No retinal changes related to high myopia and no myopic correction greater than 8.00 diopters spherical equivalent (sphere + 1 2 cylinder), No progressive ocular disease likely to affect visual acuity within the next 5 years, No lens extraction or implantation within the last 3 months, No capsulotomy within the last 3 days, No vitrectomy within the last 12 months, No LASIK surgery within the last 12 months or if the presurgical refractive error was , 8 diopters of myopia or there are pathologic retinal changes related to high myopia, No nevus . 2 disc areas within 3000 microns of the foveal center or with fluid or leakage on fluorescein angiography, Disc and macula color photographs and fluorescein angiogram within 28 days of randomization, no lens or other media opacity that would preclude good fundus photography or angiography within the next 5 years, no current use or history of using macular affecting drugs	
Reeves, 2004	People were eligible for the trial if they were newly referred to the low vision clinic at Manchester Royal Eye Hospital with a primary diagnosis of AMD. Potential participants had to have Snellen visual acuity worse than 6/18 (.0.5 logMAR) in both eyes and equal to or better than 1/60 ((1.8 logMAR) in the “better” eye.	People were ineligible if they were living in a residential or nursing home, were suffering from mental illness or dementia, or were not proficient in English
Brody, 2005	Participants in this study were recruited through ophthalmologists’ offices, the media, an AMD registry, health fairs, and senior centres. The 231 subjects were enrolled between February 1998 and September 2000. The inclusion criteria were:<br>(1) diagnosis of AMD by an ophthalmologist and confirmed by fundus photographs; (2) visual acuity of 20/60 or worse in the better eye and 20/100 or worse in the other eye with habitual correction (i.e. current glasses); (3) no other unstable eye disease or vision loss due to other eye disease; (4) age 60 years or older; (5) adequate hearing, with a hearing aid if necessary, to complete the interview and to respond in normal conversation; (6) physical ability to come to an interview if wheelchair access	

	transportation was provided; (7) no cognitive impairment as assessed by the Orientation-Memory Concentration Test[17]; and (8) no current alcohol abuse as assessed by the Short Michigan Alcoholism Screening Test.[18]	
Smith, 2005	The candidates for inclusion in the study consisted of patients referred to the low-vision clinics of the Manchester Royal Eye Hospital, Manchester, England, from July 15, 2001, through March 31, 2003, with bilateral AMD and a visual acuity (VA)of at least 1/60 (1.78 logarithm of the minimum angle of resolution [logMAR]) but no better than 6/18 (0.48 logMAR) in the better eye. To be eligible, patients had to be free of mental illness, dementia, and severe physical limitations; proficient in English; and literate; and not be a resident in a hospital or a nursing home.	
Stevenson, 2005	Patients were required to be 60 years of age or older and have evidence of subfoveal choroidal neovascularization on a fundus fluorescein angio-gram performed within 1 week of randomization. Visual acuity at baseline was required to be equal to or better than logMAR 1.0 (6/60 or better) in the study eye.	Other retinal disease, poor general health, systemic disease precluding radiotherapy
Chang, 2007	Eligible patients were 50 years or older and had a diagnosis of primary or recurrent subfoveal CNV secondary to AMD. Requirements for the study eye included an AMD lesion no larger than 12 disc areas, a neovascular component that was at least 50% of the AMD lesion, and a lesion composition that was minimally classic or occult with no classic; evidence of recent disease progression; and best corrected visual acuity of 20/40 to 20/320 (Snellen equivalent). Only 1 eye per patient was chosen as the study eye and only the study eye received either ranibizumab or sham injection. If both eyes of the patient were eligible for enrolment, the evaluating physician (along with the patient) determined which eye would be designated the study eye.	
Lamoureux,	All participants needed to be 50 years or older. Patients with visually significant	Referred patients were excluded if they had end-stage AMD in the

2007	cataract and either bilateral early AMD fulfilling the AREDS criteria for category 3 or one eye with advanced AMD and the other (study eye) with AREDS category 2 features or 3 AMD changes26were involved in the study. Patients under category 2 had mild AMD with multiple small drusen, single, or non-extensive intermediate drusen (62 to 124m), pigment abnormalities, or any combination of these in one or both eyes. Patients under category 3 had no advanced AMD in both eyes and one eye with VA20/32 with at least one large druse (125m).26Cataract status in these patients needed to be such that adequate clinical and angiographic documentation of the fundus could be achieved.	study eye or were allergic to fluorescein. Other significant ocular conditions which excluded participants from the study were high myopia (8 D sphere), diabetic retinopathy, an intermediate or posterior intraocular inflammatory disease, previous laser treatment for any retinal pathology, or the presence of diseases associated with CNV other than AMD.
Lüke, 2007	Inclusion criteria<br>Age>50 years, Age-related macular degeneration, Subfoveal CNV, Classic CNV>50% of the total lesion, Visual acuity 20/200–20/40 CNV <4 DA, Recent disease progress (decrease of the distance or reading vision within 3 months before the presentation), signed informed consent	
Rovner, 2007	The inclusion criteria were being older than 64 years and having neovascular AMD in one eye diagnosed within the preceding 6 months and preexisting AMD in the fellow eye. We chose these parameters to identify nondepressed patients with recent bilateral visual impairment who were at high risk for depression. These risk characteristics define a selective preventive intervention, which, according to the Institute of Medicine's recent classification of preventive interventions, targets a population sub-group whose biological, psychological, or social characteristics increase their risk of disorder. By contrast, an indicated preventive intervention targets higher-risk persons who already have detectable signs of disorder but do not meet diagnostic criteria.	We excluded patients with DSM- IV –defined diagnoses of depressive disorders or current treatment for depression. Many such patients were excluded prior to the baseline assessment based on medical record evidence of depression. This accounts for the low rate of depression at the baseline assessment. We also excluded patients with cognitive impairment or confounding eye conditions. Thomas Jefferson University's institutional review board approved the informed consent form and procedures to access medical records to identify eligible subjects.
Leys, 2008	Eligibility criteria included (1) age=>50years, (2) presence of subfoveal sites of choroidal neovascularization (CNV) secondary to AMD, (3) best-corrected study eye	

	visual acuity range of 20/40to 20/320, and (4) best-corrected fellow eye visual acuity of 20/800 or better. Patients with all angiographic subtypes were enrolled. Lesions could not exceed 12total disc areas (including blood, scar or atrophy, and neovascularization), of which at least 50% had to be active CNV. No more than 50% of the lesion could be a subretinal haemorrhage.	
Bressler, 2009	Only 1 eye per patient was chosen as the study eye, and only the study eye received ranibizumab with sham PDT or sham injection with active PDT. If both of a patient's was selected for treatment unless, for medical reasons, the investigator deemed the eye with worse visual acuity more appropriate for the study.<br><br>From Brown et al.: For inclusion in the study, patients had to be at least 50 years of age; have a lesion whose total size was no more than 5400 μm in greatest linear dimension in the study eye; have best-corrected visual acuity of 20/40 to 20/320 (Snellen equivalent), assessed with the use of Early Treatment Diabetic Retinopathy Study (ETDRS) charts; have no permanent structural damage to the central fovea; and have had no previous treatment (including verteporfin therapy) that might compromise an assessment of the study treatment. No patients were excluded because of preexisting cardiovascular, cerebrovascular, or peripheral vascular conditions.	Prior treatment with verteporfin, external -beam radiation therapy, or transpupillary thermotherapy in the study eye. Treatment with verteporfin in the non-study eye less than 7 days preceding day 0. Previous participation in a clinical trial (f or either eye) involving antiangiogenic drugs (pegaptanib, ranibizumab, anecortave acetat e, protein kinase C inhibitors, etc.) Previous intravitreal drug delivery (e.g., intravitreal corticosteroid injection or device implantation) in the study eye. Previous subfoveal focal laser photocoagulation in the study eye. Laser photocoagulation (juxtafoveal or extrafoveal) in the study eye within 1 month preceding day 0. History of vitrectomy surgery in the study eye
Odergren, 2010	(i)age 50 years or older; (ii) occult CNV under the geometric center of the fovea[occult CNV was defined either as a fibrovascular retinal pigment epithelium detachment (PED) or as late leak-age of undetermined source]; (iii) lesion size less than 5000lm in the greatest linear dimension (GLD); (iv) best-corrected Early Treatment Diabetic Research Study (ETRDS) visual acuity(BCVA) corresponding to a Snellen equivalent of 20⁄200 to 20⁄25; and (v)clear ocular media	
Richer, 2011	Inclusion criteria<br>We recruited patients from the DVA Medical Center Eye Clinic, with early and	Exclusion criteria<br>Subjects were excluded if they had either high-risk retinal

	moderate AMD retinopathy. These patients had symptoms and measurable deficits on the contrast sensitivity chart or demonstrated glare disturbances, Amsler grid abnormalities, or subjective functional night driving or reading disturbances that they wished to improve. The inclusion criteria used in this ZVF study are identical to those used in the Lutein Antioxidant Supplementation Trial RCT.	characteristics for advanced AMD or if they manifested advanced AMD for which existing medical or surgical options were available. Such patients typically required an AREDS I NEI Preser Vision (B 1 L Global Pharmaceuticals, Madison, New Jersey) type formula to protect the un- involved or less-involved retina in combination with anti–vascular endothelial growth factor agents. Disqualifying retinal characteristics included presence of significant active exudative AMD pathology by fluorescein angiography or optical coherence tomography (OCT), but also a single large drusen, 15 multiple intermediate drusen, parafoveal geographic atrophy, or loss of vision in 1 eye because of advanced AMD. 5 Additional exclusion criteria included consumption of L (or Zx) beyond the minimal 250 m g/d commonly found in pabulum-type daily multivitamins within 6 months. Patients known to suffer from active co- morbidities, such as uncontrolled and severe diabetes, glaucoma, uveitis, or optic neuritis, or subjects with Alzheimer's disease or non-Alzheimer's dementia or schizophrenia were excluded. Subjects using retinotoxic medications were also excluded (see the ZVF
Heier, 2012	Age 50 years with active subfoveal CNV lesions (any subtype) secondary to AMD; juxtafoveal lesions with leakage affecting the fovea also were allowed; (2) CNV comprising at least 50% of total lesion size; and (3) BCVA between 73 and 25 Early Treatment Diabetic Retinopathy Study chart (ETDRS) letters (20/40 –20/320 Snellen equivalent).	Patients with prior treatment for AMD (including an investigational agent or anti-VEGF therapy) in the study eye were excluded.
Piermarocchi, 2012	Inclusion criteria: Signed written consent, Between the ages of 55 and 80, Any race or gender, Diagnosis of nonexudative (dry) age-related macular degeneration (AMD)	Exclusion criteria<br>Ocular

	in at least one eye having extensive (as measured by drusen area) intermediate (≥63 mm, <125 mm) drusen; and at least one large (≥125 mm) drusen or geographic atrophy not involving the center of the macula, Best-corrected visual acuity in the trial eye≥20/32 (0.2 logarithm of the minimum angle of resolution [logMAR]), 74 letters of Early Treatment Diabetic Retinopathy Study [ETDRS] chart), Able to understand and comply with the requirements of the trial, Subjects must not have conditions that limit the view to the fundus (e.g., vitreous hemorrhage, cataracts, epiretinal membrane), Subjects must be available for a minimum trial duration of approximately 6 months, Subjects must agree to take only the nutritional supplement that is provided during this study, Subjects or eyes must not meet any of the exclusion criteria	Visual acuity <20/32 (VA score <74 letters ETDRS chart), Patients with advanced AMD in one or both eyes, Patients with ocular disease that causes irreversible reduction of visual acuity (amblyopia, uncontrolled glaucoma, anterior ischemic optic neuropathy, clinically significant macular edema), Patients with significant opacity of the dioptrical media that prevents fundus examination; patients with evolved cataract; all patients with lens opacity and score 4+ (Lens Opacity Classification System II) will be excluded from the enrollment, while the possible surgery has to be performed at least 2 months before the enrollment in the study, Patients with insufficient pupil dilation, Patients already subjected to laser treatment of the posterior pole for any other reason, Patients with macular changes not attributable to AMD<br>General<br>Patients with carotenoids intolerance, Patients with major chronic disease, Life expectation lower than 6 months, Withdrawal of informed consent, Enrollment in another clinical study with experimental product within the last 4 weeks or during the current study, Patients unable and/or not willing
Brunner, 2013	Fifty-four eyes of 54 patients with cataract and nonexudative AMD were consecutively enrolled at the outpatient care department of the Department of Ophthalmology, Rudolph Foundation Clinic, Vienna, Austria.	Patients with retinal pathologies other than AMD, exudative AMD, progressive glaucoma, myopia > 10 diopters, panretinal laser treatment, former cataract or vitreoretinal surgery were excluded.
Mielke, 2013	Eingeschlossen wurden alle Patienten mit AMD, die sich zur Erstversorgung mit Hilfsmitteln in der Sehbehindertenambulanz Tübingen vorstellten. Die Patienten sollten zwischen 65 und 85 Jahre alt sein. Der bestkorrigierte Visus auf dem besseren Auge sollte 0,05–0,2 betragen, um einerseits zu gewährleisten, dass eine	Ausgeschlossen wurden Patienten mit schweren motorischen Störungen, die die Handhabung von Hilfsmitteln nicht ermöglicht hätten, Patienten mit manifester Demenz, schwerer Depression so wie mit psychotischen Symptomen oder Suizidalität, da Letztere

	Wiederherstellung der Lesefähigkeit potenziell möglich war, zum anderen aber auch, dass tatsächlich ein Versorgungsbedarf mit vergrößernden Sehhilfen bestand.	akut antidepressiv behandelt werden sollte.
Rovner,2013	The inclusion criteria were: 1) age 65 years or older; 2) bilateral AMD (neovascular and/or geographic atrophy); 3) visual acuity between 20/70 and 20/400 [inclusive; (best corrected)] in the better-seeing eye, and no lower acuity limit in the fellow eye; and 4) moderate difficulty in at least one valued vision-function goal (e.g., reading mail, attending social activities).	The exclusion criteria were: 1) presence of uncontrolled glaucoma, diabetic retinopathy, or planned cataract surgery within 6 months; 2) cognitive impairment on an abbreviated version of the Mini-Mental Status Examination (MM blind) that omits vision-dependent items; 16 3) presence of a medical condition that would preclude participation; and 4) residence in a skilled nursing facility.
Rovner, 2014	The inclusion criteria were: (1) aged over65 years; (2) bilateral AMD (either neo-vascular or "wet" AMD) or geographic atrophy (that is, "dry" AMD); (3) best corrected visual acuity worse than 20/70in the better-seeing eye; (4) more than five anti-vascular endothelial growth fac-tor (VEGF) injections if the better eye had "wet" AMD; (5) subthreshold depressive symptoms (defined as a Patient HealthQuestionnaire-9 score5 or depressed mood or anhedonia several days per week; and (6) moderate difficulty performing a valued vision-dependent activity.	The exclusion criteria were: (1) on- going or anticipated anti-VEGF treatment; (2) current Diagnostic and Statistical Manual (DSM) IV– defined depressive disorder; (3) uncontrolled glaucoma, diabetic retinopathy, corneal dystrophy, or anticipated cataract surgery; (4) low vision rehabilitation within the previous year; and (5) cognitive impairment on an abbreviated version of the Mini-Mental Status Examination (MM blind) that omits vision-dependent items (Reischies & Geiselmann, 1997).
Huang, 2015	Subjects with AMD aged over 50 years were recruited in Beijing, China. Inclusion criteria included a clinical diagnosis of early AMD (defined as the presence of soft drusen, presence of retinal pigmentary abnormalities with no signs of late AMD, or both) according to the Age-Related Eye Disease Study System [17], clear ocular media, and agreement to adhere to the study regimen.	Those who had other ocular disorders or unstable systemic or chronic illness or consumed dietary supplements containing antioxidants or carotenoids within the previous 6 months were excluded. This study was performed in accordance with the principles of the Declaration of Helsinki and was approved by the Medical Ethics Committee of Peking University. Written consent was obtained from all subjects.
Markun, 2015	Eligible subjects were male or female patients, aged 50 years or older, with W-AMD, who were eligible for therapy with antiangiogenic drugs, had a best corrected visual	Exclusion criteria were serious general or psychological illness (advanced malignant diseases, severe depressive disorders or

	acuity of at least 20 letters assessed with the Early Treatment Diabetic Retinopathy (ETDRS) chart and provided written informed consent in study participation. In cases where both eyes were affected by W-AMD, both eyes were included and followed in the study.	dementia) and insufficient German or French language skills (for completing the self-administered questionnaires). We intended to include treatment-naïve patients, thus patients with any former invasive medical treatment for W-AMD were also excluded.
7, 2016	Inclusion criteria: Participants over the age of 50 with dry type ARMD who were admitted to the ophthalmology clinic at the Baqiyatallah Hospital (Tehran, Iran) were included in this study.	Exclusion criteria: The following cases were excluded from the study; wet and severe dry type ARMD and patients with systemic diseases such as hypertension, diabetes, or glaucoma, ARMD secondary to retinal diseases, and patients taking any other dietary supplements. .
Tao, 2016	Dry AMD	AMD was diagnosed according to a complete ophthalmic examination and met the following criteria: (1) No diabetes or hypertension that may affect to retinal function; (2) Lens opacity and ocular media remained transparent; (3) No family history of glaucoma, IOP was normal and C/D ≤ 0.4; (4) No high myopia, uveitis and retinal detachment which may affect the macular function.
Akuffo, 2017	Inclusion criteria for the trial were as follows: nonadvanced AMD (1 to 8 on the AREDS 11-step severity scale12 in at least one eye [the study eye], confirmed by the Moorfields Eye Hospital Reading Centre, London, UK, an accredited retinal grading center); best-corrected visual acuity (BCVA) of 6/12 (20/40) or better in the study eye; no more than five diopters spherical equivalent refraction in the study eye; no previous consumption of supplements containing the macular carotenoids (L and/or Z and/or MZ); no retinal pathology other than AMD; and no diabetes mellitus (by self-report). The study eye could be either the right or left eye. If both eyes exhibited nonadvanced AMD, the eye with the best BCVA was chosen as the study eye. However, if each eye had the same BCVA and nonadvanced AMD, the right eye was	

	selected. Each participant provided written informed consent of their willingness to participate in the trial, and the examination procedures adhered to the tenets of the Declaration of Helsinki	
Lek, 2017	Enrollment was restricted to people aged 50 to<95 years with lesion characteristics that met the criteria for intermediate AMD as defined by the Beckman classification.17 In the Beckman classification, intermediate AMD is defined as large (>125mm)drusen or any AMD pigmentary abnormalities that are associated with medium (>63mm) or large drusen within 2 disc diameters(w3000mm) from the fovea. To select intermediate AMD cases with a greater risk of progression, our inclusion criteria required at least one>125mm druse to be within 1500mm from the fovea in both eyes based on color fundus photography (CFP)(Ferris FL, personal communication, November 20, 2012). In addition, participants were required to have a repeatable relative scotoma (<25 decibels [dB], 2 standard deviations fromcontrols18) in at least 1 location (same or adjacent) within a central 6grid of the Macular Integrity Assessment micro perimeter in 1 eye. This criterion is consistent with a previous study that reported reduced sensitivity as a risk factor for progression to GA19and increases the likelihood of detecting any improvement in macular function. Eligible participants were required to have best-corrected visual acuity (BCVA) of20/40in both eyes, and no ocular, systemic, or neurologic disease that could compromise retinal assessment.	Individuals with late AMDe CNV and CFP-defined GA (Table 2 and Fig 1A and B) were excluded. In addition, we excluded anyone without signs of GA on CFP but who had evidence of anatomic changes on MMI that potentially portend the development of GA.20,21 These MMI signs included fundus autofluorescence (FAF)-defined atrophy,22 spectral-domain OCT (SD-OCT) signs of atrophy,20 and nascent geographic atrophy (nGA)21 (Table 2 and Fig 1CeH). We also excluded individuals with subfoveal large (>1000 mm) drusenoid pigment epithelial detachment, because our pilot study suggested that these individuals had a higher risk of progression to GA despite treatment.11
Lee, 2018	Participants (age ≥ 50 years) with a diagnosis of symptomatic macular PCV (greatest linear dimension of lesion, <5400mm or ~ 9 Macular Photocoagulation Study disc areas) were included; an active PCV diagnosis and study eligibility were investigator determined. Participants must also have had a best-corrected visual acuity (BCVA) of 73 to 24 Early Treatment Diabetic Retinopathy Study (ETDRS) letters (20/40-20/320 Snellen) in the study eye. Full inclusion/exclusion criteria are provided in	Prior use of intravitreal or sub-tenon corticosteroids in the study eye within 3 months prior to study entry<br>2. Any prior use of intraocular anti-VEGF agents in the study eye, or systemic use of anti-VEGF products within 3 months prior to study entry<br>3. Prior macular laser treatment in the study eye including PDT

	eTable1 in Supplement 1.	4. Only 1 functional eye (a functional eye is defined as one that is not legally blind) even if that eye is otherwise eligible for the study. Furthermore, subjects with only 1 eligible eye should not have other ocular conditions with poorer prognosis in the fellow eye<br>5. Any ocular disorders in the study eye that, in the opinion of the investigator, may confound interpretation of study results or interfere with subject safety, including, but not limited to:<br>a. Significant media opacities, including cataract, which can interfere with visual acuity, or fundus photography<br>b. Significant scarring or atrophy in the macula that indicates substantial irreversible vision loss, or other conditions limiting capacity for visual acuity improvement<br>c. Decrease in BCVA due to causes other than wet AMD/PCV<br>d. History or presence of diabetic macular edema or diabetic retinopathy<br>e. Vitreomacular traction or epiretinal membrane in the study eye evident biomicroscopically or on OCT that is considered by the investigator to significantly affect central vision<br>f. Ocular inflammation (including trace or above) or external ocular inflammation in the study eye. Evidence at examination of infectious blepharitis, keratitis, scleritis, or conjunctivitis in either eye or current treatment for serious systemic infection<br>g. History of idiopathic or autoimmune uveitis in either eye<br>h. Aphakia or absence of the posterior capsule; with the exception of pseudoaphakia or yttrium aluminum garnet (YAG) capsulotomy in the study eye

		6. Concomitant systemic disease which may affect the patient´s regular visits or contraindicate use of PDT including, but not limited to:<br>a. Uncontrolled hypertension defined as a single measurement of systolic >180 mm Hg, 2 consecutive measurements of systolic >160 mm Hg, or 1 measurement of diastolic >100 mmHg on optimal medical regimen<br>b. Uncontrolled diabetes mellitus, in the opinion of the investigator<br>c. History of cerebrovascular disease or myocardial infarction within 6 months prior to entry into the study<br>d. Renal failure requiring dialysis or renal transplant<br>e. Clinically relevant impairment of liver function, in particular porphyria<br>7. Participation in an investigational study within 30 days prior to the initial screening visit that involved treatment with any drug (excluding vitamins and minerals) or device<br>8. Pregnancy or lactation<br>9. History of allergy to fluorescein used in fluorescein angiography, iodine and/or indocyanine green<br>10. History of allergy to aflibercept, verteporfin, or their excipients
Kaltenegger, 2019	Inclusion criteria: Dry AMD with an absolute central scotoma including the foveola, (small parafoveal seeing islands were permitted), non-foveal (parafoveal or eccentric) fixation, magnification requirement 1.25 – 10-fold, visual acuity 0.2 – 1.1 logMAR (decimal 0.08 – 0.60), previous adaptation to the use of magnifying aids of at least 6 weeks.	Exclusion criteria: Any other eye disease, exudative AMD, simultaneous anti-VEGF or other treatments, markedly reduced emotional and cognitive status that could have jeopardized participation in the study. The better eye had to fulfill the inclusion criteria.
Heier, 2020	In brief, the studies included participants 50 years of age or older with bilateral GA	

	secondary to AMD and without evidence of active or prior, or previous treatment for, choroidal neo-vascularization in either eye. One eye was selected as the study eye, which met the following key inclusion criteria: total GA lesion size (unifocal or multifocal) of 2.54 to 17.78 mm2(1e7 disc areas)measured on blue-light fundus autofluorescence (FAF), with perilesional banded or diffuse autofluorescence patterns, and Early Treatment Diabetic Retinopathy Study (ETDRS) best-corrected visual acuity (BCVA) letter score of 49 or more (Snellen equivalent, 20/100 or better). In study eyes with BCVA letter score of 79or more (Snellen equivalent, 20/25 or better), at least 1 lesion was required within 250mm of the foveal center. Further details on inclusion criteria and study eye selection are provided in the prior publication from these studies.<br><br>From Holz et al.: If both eyes were eligible, the eye with the poorer visual function as determined by the investigator and the patient was selected, followed by the eye with the larger GA lesion. Participants were also evaluated at screening for CFI-profile genetic biomarker status (eTable 2 in Supplement 3).	
Kunimoto, 2020	Patients at least 50 years of age with treatment-naïve AMD were enrolled. Study eyes were required to have active subfoveal or juxtafoveal CNV (within 200 mm from foveal center) secondary to AMD evidenced by leakage on fluorescein angiography, CNV lesion area comprising >50% of the total lesion area as assessed by the investigator and confirmed by the central reading center (CRC) (University of Wisconsin Fundus Photography Reading Center, Madison, WI), and best-corrected visual acuity (BCVA) between 73 and 24 Early Treatment Diabetic Retinopathy Study letters (20/40 to 20/320 Snellen equivalent).<br><br>If both eyes were eligible for the study, the eye with worse BCVA was selected as	Patients with any previous treatment for nAMD in the study eye (except dietary supplements or vitamins) were excluded. Other key exclusion criteria included active periocular, ocular, or intraocular infection at baseline; history of recurrent or currently active ocular or intraocular inflammation (IOI) (e.g., uveitis) at baseline; history or clinical evidence of diabetic retinopathy, diabetic macular edema, or any retinal vascular disease other than AMD at screening; prior use of ocular anti-VEGF agents for neovascular eye diseases other than AMD in the study eye; and history of pars plana vitrectomy, submacular surgery, or incisional glaucoma

	the study eye. If both eyes were eligible and had identical BCVA values, the patient could elect to have the nondominant eye selected for study treatment, and if the patient did not do so, the right eye was selected as the study eye.	surgery in the study eye. A complete listing of the inclusion and exclusion criteria is provided in Table S1 (available at www.aaojournal.org).
Visser, 2020_1	To be eligible, patients had to be at least 65 years old, have a best-corrected visual acuity of 20/200 to 20/20 (Snellen equivalent) in the study eye as assessed using Early Treatment Diabetic Retinopathy Charts (ETDRS), no previous ARMD treatment and active leakage. Patients were only treated in one eye. Fluorescein angiography (FA) and indocyanine green (ICG) angiography were used to observe leakage, and optical coherence tomography (OCT) was used to observe the presence of fluid [26].	Patients who had other significant ocular disorders, had allergies to either FA or ICG dye injections, were immunocompromised, using coumarin-derivatives, had experienced a clinically significant cerebrovascular accident or myocardial infarction or had a planned ocular surgery during the 1-year follow-up, were excluded.
Visser, 2020_2	Inclusion criteria were as follows: (1) previously diagnosed with AMD and referral by an ophthalmologist, (2) age older than 18 years,(3) reported benefit from E-Scoop during the initial baseline visit, and (4) a signed informed consent form.	inability to understand Dutch or physical/cognitive impairment precluding testing
Gabrielle, 2023	Eligible patients were at least 50 years of age and demonstrated visual loss resulting from a recent SMH (first symptoms14 days before treatment) secondary to nAMD, with (1) the presence of blood predominantly overlying the retinal pigment epithelium(RPE) with a minimum thickness of more than 100mm assessed through spectral-domain (SD) OCT and its manual caliper function and (2) SMH diameter more than 2 optic disc diameters (DD) on retinal photographs. Only 1 eye per patient was included.	Patients were excluded if they had any of the following: SMH linked to a cause other than AMD (such as myopia, angioid streaks, or arterial macroaneurysm), history of SMH in the same eye, presence of macular scar, patient seeking treatment more than 14 days after the onset of visual loss, sub-RPE hemorrhage exclusively, thin hemorrhage without retinal elevation (maximum thickness of the clot < 100 m m on SD OCT), international normalized ratio for coagulation of more than 4 (contraindicating surgery), or need for cataract surgery within the first 3 months of study.
Tanveer, 2023	Patients aged >50 years with age-related macular degeneration, patients of both genders (male and female), participants willing to give informed consent for participation in the study	Presence of any ocular disease or systemic ocular pathology impairing visual function, presence of disorders causing choroidal neovascularization other than age-related macular degeneration,

		patients with mental illness, dementia, or severe physical limitations
Boyer, 2024	Subjects were eligible for enrollment (NCT04065490) if they were at least 50 years and had a diagnosis of dry AMD defined by the presence of drusen and/or nonfoveal center GA with best corrected visual acuity (BCVA) scores determined by Early Treatment Diabetic Retinopathy Study (ETDRS) between 50 and 75 (Snellen equivalent: 20/ 32 to 20/100) (see Table, Supplemental Digital Content 1, http://links.lww.com/IAE/C119).	Subjects were excluded with a history of CNV, presence of center involving GA, or other significant retinal disease. Each eye was individually assessed for drusen, GA, and CNV by a central reading center (Duke Reading Center, Durham, NC).19
Jackson, 2024	Eligible participants were aged 50 years or older and had chronic active nAMD, with at least three previous anti-VEGF injections, including at least one in the last 4 months; if two eyes were eligible, the participant chose one as the study eye. Study eyes had to require an injection at the time of enrolment, with a macular volume 8·15 mm^3 or greater (varying by machine), measured using spectral-domain optical coherence tomography (SD-OCT).	People with diabetes were excluded, alongside patients in whom the study eye had previous nAMD treatment other than anti-VEGF therapy; those with foveal scarring; an Early Treatment Diabetic Retinopathy (ETDRS) BCVA letter score worse than 24 (6/96); Study15 a lesion diameter greater than 4 mm, or extending more than 2 mm from the foveal center. Full exclusion criteria are shown in the appendix (pp 22–23). Baseline ocular imaging was sent to an independent reading center but, for pragmatic reasons and to enhance generalizability, eligibility was determined by the attending ophthalmologist (appendix pp 22–23).
Lanzetta, 2024	Adults aged 50 years or older at randomization were eligible to participate in the study, with one eye per patient designated as the study eye. Active, subfoveal choroidal neovascularisation secondary to nAMD (including juxtafoveal lesions that affect the fovea), best-corrected visual acuity (BCVA) according to an Early Treatment Diabetic Retinopathy Study (ETDRS) letter score of 78−24 (corresponding to a Snellen equivalent of approximately 20/32 to 20/320), and total area of choroidal neovascularisation (including both classic and occult components) of more than 50% of the total lesion area were required in the study eye. The total	Exclusion criteria included total lesion size of more than 12 disc areas (30·5 mm^2) in the study eye. Eligibility of the study eye was confirmed by the central reading center (appendix pp 9−11). All

	lesion area was measured by including all lesion components such as choroidal neovascularisation, blocked fluorescence, thick contiguous blood, haemorrhagic and serous pigment epithelium detachment, retinal pigment epithelium rip or tear, fibrous tissue, non-fibrous scar, and macular atrophy contiguous to the choroidal neovascularisation lesion or developing in the place where choroidal neovascularisation was previously present. Intraretinal fluid, subretinal fluid, or both affecting the center subfield of the study eye must have been observed on spectral-domain optical coherence tomography (SD-OCT), for which the center subfield was defined as a circle with a 1 mm diameter, centered on the fovea.	

AMD = Age-related macular degeneration; anti-VEGF = Anti-vascular endothelial growth factor; AREDS = Age-Related Eye Disease Study; ARMD = Age-related macular degeneration; BCVA = Best-corrected visual acuity; C/D = Cup-to-disc ratio; CFI = Complement factor I; CFP = Color fundus photography; CNV = Choroidal neovascularization; CRC = Central reading center; D = Diopter; DA = Disc area; dB = Decibel; DD = Disc diameter; DSM-IV = Diagnostic and Statistical Manual of Mental Disorders, Fourth Edition; DVA = Department of Veterans Affairs; ETDRS = Early Treatment Diabetic Retinopathy Study; FA = Fluorescein angiography; FAF = Fundus autofluorescence; GA = Geographic atrophy; GLD = Greatest linear dimension; ICG = Indocyanine green angiography; IOI = Intraocular inflammation; IOP = Intraocular pressure; L = Lutein; LASIK = Laser-assisted in situ keratomileusis; logMAR = Logarithm of the minimum angle of resolution; MM blind = Mini-Mental Status Examination, blind version; MMI = Multimodal imaging; MPS = Macular Photocoagulation Study; MZ = Meso-zeaxanthin; nAMD = Neovascular age-related macular degeneration; nGA = Nascent geographic atrophy; NEI = National Eye Institute; OCT = Optical coherence tomography; PCV = Polypoidal choroidal vasculopathy; PDT = Photodynamic therapy; PED = Pigment epithelial detachment; RCT = Randomized controlled trial; RPE = Retinal pigment epithelium; SD-OCT = Spectral-domain optical coherence tomography; SMH = Submacular hemorrhage; VA = Visual acuity; VEGF = Vascular endothelial growth factor; W-AMD = Wet age-related macular degeneration; YAG = Yttrium aluminum garnet; Z = Zeaxanthin; ZVF = Zeaxanthin and Visual Function Study; Zx = Zeaxanthin.
