# Supplementary material for: Vision-Related Patient-Reported Outcomes in Randomized Controlled Trials of Age-Related Macular Degeneration: A Systematic Review
Source: Ophthalmol Sci. 2026 Jun 30;6(9):101308. doi: 10.1016/j.xops.2026.101308 (PMC13449777; doi:10.1016/j.xops.2026.101308)
Supplement: Supplementary file 3 [file mmc3.pdf]

Supplementary Table 2 - Detailed Quality Assessment

	Signaling Questions*									Risk of Bias Assessment		
Author, Year	1.1 Random Sequence Generation	1.2 Allocation Concealment	1.3 Baseline Imbalance	3.1 Outcome data Available	3.2 Not Biased by Missing Data	3.3 Missingness Depends on True Value	3.4 Missingness Likely Depends on True Value	4.1 Outcome Measurement Appropriate	4.2 Outcome Measurement Differs by Group	Domain 1	Domain 3	Domain 4
Brody, 2001	No information	No information	No	No information	Probably no	No information	No information	No	No	Some concerns	High risk	Low risk
Dong, 2004	Yes	Yes	No	Yes				No	No	Low risk	Low risk	Low risk
Maguire, 2004; Ying, 2008	Yes	Yes	No	Yes				No	No	Low risk	Low risk	Low risk
Reeves, 2004	Yes	Yes	No	Yes				No	No	Low risk	Low risk	Low risk
Brody, 2005	Yes	Yes	No	No	Probably no	No information	No information	No	No	Low risk	High risk	Low risk
Smith, 2005	Yes	Probably yes	No	Probably yes				No	No	Low risk	Low risk	Low risk
Stevenson, 2005	Probably yes	Yes	No	Yes				No	No	Low risk	Low risk	Low risk
Chang, 2007	Yes	Yes	No	Yes				No	No	Low risk	Low risk	Low risk
Lamoureux, 2007	No information	No information	Probably yes	No	Probably no	Probably no	Probably no	No	No	High risk	Low risk	Low risk
Lüke, 2007	Probably yes	Probably yes	Probably no	Probably yes				No	No	Low risk	Low risk	Low risk
Rovner, 2007	Yes	Probably yes	No	Yes				No	No	Low risk	Low risk	Low risk
Leys, 2008	Yes	Probably yes	No	Yes				No	No	Low risk	Low risk	Low risk

	Signaling Questions*									Risk of Bias Assessment		
**Author, Year**	**1.1 Random Sequence Generation**	**1.2 Allocation Concealment**	**1.3 Baseline Imbalance**	**3.1 Outcome data Available**	**3.2 Not Biased by Missing Data**	**3.3 Missingness Depends on True Value**	**3.4 Missingness Likely Depends on True Value**	**4.1 Outcome Measurement Appropriate**	**4.2 Outcome Measurement Differs by Group**	**Domain 1**	**Domain 3**	**Domain 4**
Bressler, 2009	Probably yes	Probably yes	No	Yes				No	No	Low risk	Low risk	Low risk
Odergren, 2010	No information	No information	No	Yes				No	No	Some concerns	Low risk	Low risk
Richer, 2011	Yes	Yes	Probably no	Yes				No	No	Low risk	Low risk	Low risk
Heier, 2012	Yes	Yes	No	Yes				No	No	Low risk	Low risk	Low risk
Piermarocchi, 2012	Probably yes	Probably yes	No	Yes				No	No	Low risk	Low risk	Low risk
Brunner, 2013	No information	No information	No	No	Probably no	No information	No information	No	No	Some concerns	High risk	Low risk
Mielke, 2013	No information	No information	Probably no	Probably no	Probably yes			No	No	Some concerns	Low risk	Low risk
Rovner,2013	Yes	Yes	No	Yes				No	No	Low risk	Low risk	Low risk
Rovner, 2014	Yes	Probably yes	No	Probably yes				No	No	Low risk	Low risk	Low risk
Huang, 2015	Yes	No information	No	Probably yes				No	No	Some concerns	Low risk	Low risk
Markun, 2015	Yes	Yes	No	Yes				No	No	Low risk	Low risk	Low risk
Riazi, 2016	No information	No information	Probably yes	No	No	Probably yes	No information	No	No	High risk	High risk	Low risk
Tao, 2016	No information	No information	No	Yes				No	No	Some concerns	Low risk	Low risk

	Signaling Questions*									Risk of Bias Assessment		
**Author, Year**	**1.1 Random Sequence Generation**	**1.2 Allocation Concealment**	**1.3 Baseline Imbalance**	**3.1 Outcome data Available**	**3.2 Not Biased by Missing Data**	**3.3 Missingness Depends on True Value**	**3.4 Missingness Likely Depends on True Value**	**4.1 Outcome Measurement Appropriate**	**4.2 Outcome Measurement Differs by Group**	**Domain 1**	**Domain 3**	**Domain 4**
Akuffo, 2017	Yes	Yes	No	Yes				No	No	Low risk	Low risk	Low risk
Lek, 2017	Yes	Yes	No	Yes				No	No	Low risk	Low risk	Low risk
Lee, 2018	Yes	Yes	No	Yes				No	No	Low risk	Low risk	Low risk
Kaltenegger, 2019	No information	No information	No information	No information	Probably no	No information	No information	No	No	Some concerns	High risk	Low risk
Heier, 2020	Probably yes	Probably yes	No	No	Probably no	No information	No information	No	No	Low risk	High risk	Low risk
Kunimoto, 2020	Yes	Yes	No	Yes				No	No	Low risk	Low risk	Low risk
Visser, 2020_1	Yes	No information	No	Yes				No	No	Some concerns	Low risk	Low risk
Visser, 2020_2	Yes	Probably yes	No	Yes				No	No	Low risk	Low risk	Low risk
Gabrielle, 2023	Yes	Probably yes	No	Yes				No	No	Low risk	Low risk	Low risk
Tanveer, 2023	Probably no	Probably no	No	Yes				No	No	High risk	Low risk	Low risk
Boyer, 2024	Probably yes	Yes	No	Yes				No	No	Low risk	Low risk	Low risk
Jackson, 2024	Yes	Yes	No	Yes				No	No	Low risk	Low risk	Low risk
Lanzetta, 2024	Probably yes	Yes	No	Probably yes				No	No	Low risk	Low risk	Low risk

*See Supplementary Data 2 for detailed Risk of bias (RoB 2) approach and modifications
RoB = Risk of bias; RoB 2 = Risk of Bias 2.
